# TET1 participates in oxaliplatin-induced neuropathic pain by regulating microRNA-30b/Nav1.6

**DOI:** 10.1016/j.jbc.2025.108228

**Published:** 2025-01-27

**Authors:** Sen Zhao, Jing-jing Zhang, Meng-ya Zhang, Qing-qing Yang, Zhi-xiao Li, Xiu-hua Ren, Song-xue Su, Tian-en Si, Jian-min Li, Hui-rui Wu, Shi-yue Chen, Wei-dong Zang, Jing Cao

**Affiliations:** 1 Department of Human Anatomy, Histology and Embryology, School of Basic Medical Sciences, Zhengzhou University, Zhengzhou, China; 2 Institute of Neuroscience, School of Basic Medical Sciences, Zhengzhou University, Zhengzhou, China; 3 School of Nursing and Health, Zhengzhou University, Zhengzhou, China; 4 Centre for Sport Nutrition and Health, Centre for Nutritional Ecology, School of Physical Education (Main Campus), Zhengzhou University, Zhengzhou, China

**Keywords:** DRG, epigenetics, 5-hydroxymethylcytosine, microRNA, Nav1.6, oxaliplatin, pain, sodium channels, TET1

## Abstract

Chemotherapy-induced neuropathic pain poses significant clinical challenges and severely impacts patient quality of life. Sodium ion channels are crucial in regulating neuronal excitability and pain. Our research indicates that the microRNA-30b (miR-30b) in rat dorsal root ganglia (DRG) contributes to chemotherapy-induced neuropathic pain by regulating the Nav1.6 protein. Additionally, ten-eleven translocation methylcytosine dioxygenase 1 (TET1) plays a crucial role in pain generation by altering gene expression. We established a chemotherapy-induced neuropathy model using intraperitoneal oxaliplatin (OXA) injections and measured TET1 and Nav1.6 protein in the DRG. Using lentivirus and *Tet1*^*flox/flox*^ mice, we modulated TET1 expression and assessed pain behaviors, DRG neuronal excitability, Nav1.6 currents, miR-30b-5p, and demethylation of the *Mir30b* promoter region. We employed chromatin immunoprecipitation to pinpoint TET1-binding sites on the *Mir30b* promoter. The impacts of miR-30b agomir or antagomir on Nav1.6 expression and pain responses were assessed postintrathecal injections. The results showed that OXA reduced TET1, increasing neuronal excitability, Nav1.6 currents, and miR-30b-5p in the DRG. TET1 knockdown exacerbated these effects and induced pain behaviors. Conversely, TET1 overexpression reversed these effects. TET1 also targeted and enhanced demethylation at the *Mir30b* promoter (−1103 bp to −1079 bp). miR-30b agomir reduces Nav1.6, whereas miR-30b antagomir reverses TET1’s effects on Nav1.6 and pain. In OXA-induced neuropathy, decreased TET1 reduces miR-30b, elevating Nav1.6 expression and currents and contributing to pain. We hypothesize that TET1 mediates this process by regulating the demethylation of the *Mir30b* promoter.

Oxaliplatin (OXA), a third-generation platinum-based chemotherapeutic agent, is used as a first-line treatment for colorectal cancer, gastric cancer, ovarian cancer, and lymphoma (1, 2, 3). Evidence shows that OXA treatment can cause severe peripheral pain syndromes, particularly cold hyperalgesia, in nearly 90% of the patients (4, 5). However, the underlying mechanisms leading to neuropathic pain remain poorly understood. Recently, OXA has been reported to alter gene expression and cause functional changes in the dorsal root ganglia (DRG), leading to chemotherapy-induced neurotoxicity (6, 7). Therefore, targeting molecules or pathways involved in OXA-induced gene expression changes in the DRG may offer new strategies for managing neuropathic pain.

Epigenetic mechanisms like DNA methylation are crucial determinants in neuropathic pain pathogenesis, primarily responsible for gene silencing and genome stability (8). Ten-eleven translocation methylcytosine dioxygenase 1 (TET1), a DNA demethylase, initiates the DNA demethylation by converting 5-methylcytosine (5 mC) to 5-hydroxymethylcytosine (5hmC) in the CpG dinucleotide, leading to increased gene expression (9, 10, 11, 12). Studies show that TET1 contributes to neuropathic pain development by catalyzing 5 mC to 5hmC conversion in pain-associated gene promoters like brain-derived neurotrophic factor, metabotropic glutamate receptors five, and potassium voltage-gated channel subfamily A member 2 (*Kcna2*) in DRG or dorsal horn neurons, indicating a critical role in pain processing (13, 14, 15). Furthermore, spinal TET1 is implicated in OXA-induced neuropathic pain by demethylating the SRY-Box transcription factor 10 (*Sox10*) promoter in rats (16). Previous research showed that TET1 regulates trigeminal inflammatory pain by targeting Kv7.2 in mice (17). However, it is still unclear whether DRG TET1 influences nociceptive processing by demethylating pain-associated genes in OXA-induced neuropathic pain.

Most research indicates that activating voltage-gated sodium channels, including isoforms Nav1.3, Nav1.6, Nav1.7, Nav1.8, and Nav1.9, increases neuronal excitability and contributes to pain processing in primary sensory neurons (18, 19). The tetrodotoxin-sensitive (TTX-s) sodium channel Nav1.6 is implicated in the functional changes in DRG neurons postvincristine treatment (20). Recent studies have revealed an increase in Nav1.6 in DRG following OXA treatment, contributing to peripheral neuropathy and playing a key role in OXA-induced cold hyperalgesia (21). Furthermore, effectively alleviating pain by inhibiting Nav1.6 upregulation with siRNA highlights its therapeutic potential (22). Thus, Nav1.6 is a crucial target for pain management, although the mechanisms governing its upstream regulation are still unclear.

To this end, we established an OXA-induced neuropathic pain mouse model and utilized *Tet1*^*flox/flox*^ mice and gene overexpression (OE) strategies to explore TET1’s role in OXA-induced pain and its interactions with Nav1.6. Our findings indicate that TET1 upregulation reduces Nav1.6 expression and currents through increased miR-30b, providing pain relief and prompting further investigation into the underlying mechanism.

## Results

### OXA administration leads to nociceptive hypersensitivities and reduction of TET1

**Figure 1:**
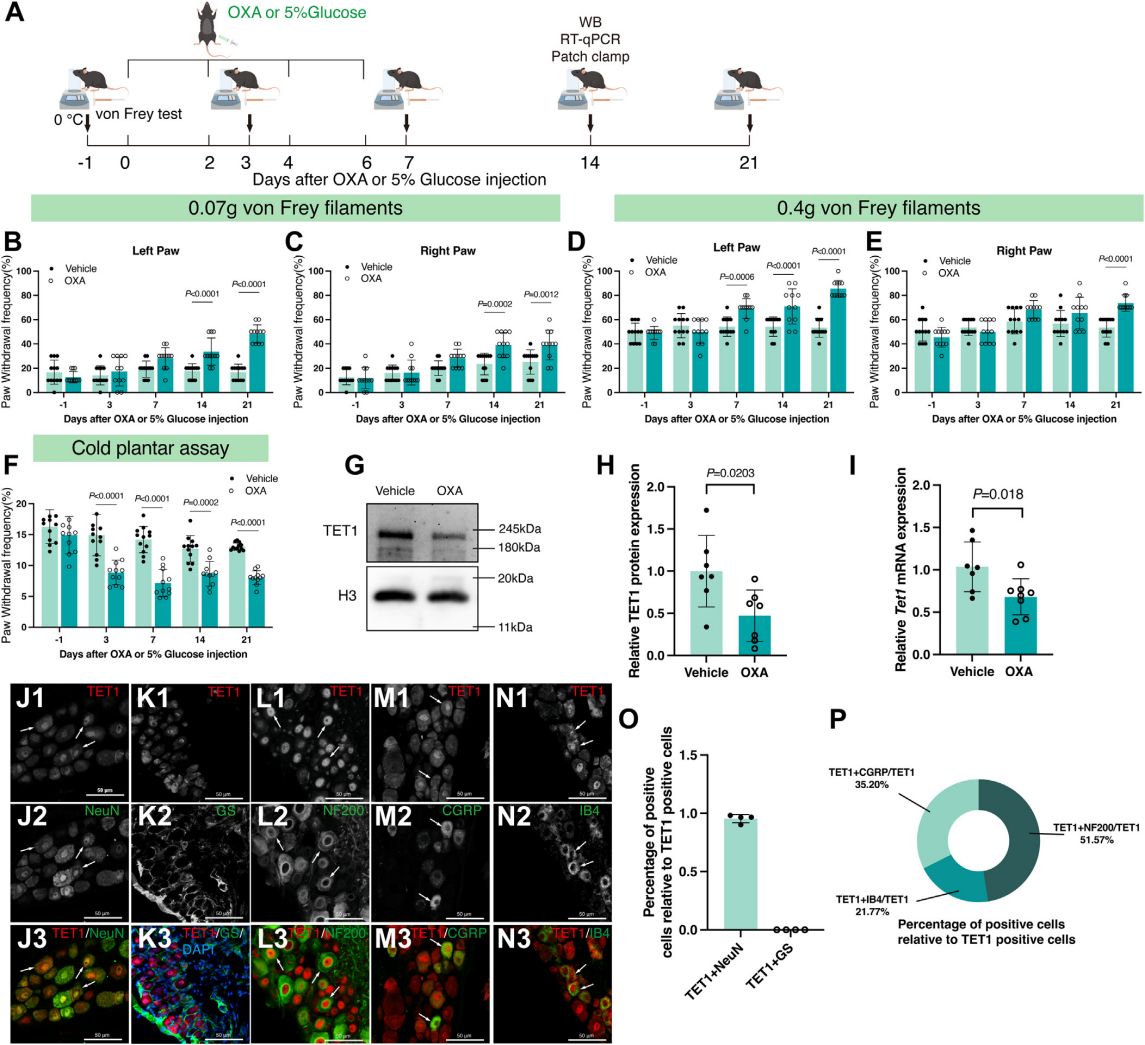
**Oxaliplatin caused mechanical allodynia, cold hyperalgesia, and downregulated TET1 expression.***A*, schematic diagram of drug injection and behavioral tests. *B* and *C*, 14 days post-OXA injection, mice in the OXA group showed mechanical allodynia upon 0.07 g von Frey filament stimulation of both hind paws, in contrast to the vehicle group (two-way ANOVA. *n* = 12). *D* and *E*, in the OXA group, the PWF for the *left* hind paw (*D*) was increased on day 7 postinjection, and for the right hind paw (*E*) on day 21, following 0.4 g von Frey filament stimulation, relative to the vehicle group (two-way ANOVA. *n* = 12). *F*, mice in the OXA group exhibited cold hyperalgesia relative to the vehicle group (two-way ANOVA. *n* = 12). *G*–*I*, on day 14 after OXA injection, the expression of TET1 protein (*G* and *H*) and mRNA (*I*) in bilateral DRG of mice decreased by about 52.83% and 31.85%, respectively (unpaired *t* test. *n* = 7–8). *J*–*N*, cells (arrows) were labeled for TET1 (*red*), NeuN (*green*), GS (*green*), NF200 (*green*), CGRP (*green*), and IB4 (*green*) in WT mice (*n* = 4). Scale bar represents 50 μm. *O*, percentage of TET1+NeuN- and TET1+GS-positive cells relative to TET1-positive cells (*n* = 4). *P*, percentage of TET1+NF200-, TET1+IB4-, and TET1+CGRP-positive cells relative to TET1-positive cells (*n* = 3 in TET1+CGRP/TET1, *n* = 6 in TET1+IB4/TET1, and *n* = 4 in TET1+NF200/TET1). CGRP, calcitonin gene-related peptide; DRG, dorsal root ganglia; GS, glutamine synthetase; IB4, isolectin B4; NeuN, neuron; NF200, neurofilament 200; OXA, oxaliplatin; PWF, paw withdrawal frequency; PWL, paw withdrawal latency; Veh, vehicle; WT, wild type.

We examined the effects of chemotherapy-induced peripheral neuropathy using a mouse model of neuropathic pain. The model was established using an intraperitoneal injection of OXA (see details in Experimental procedures and Fig. 1*A*). The von Frey test and cold plate assay were performed at −1, 3, 7, 14, and 21 days after the first OXA administration to confirm the successful establishment of the model (Fig. 1*A*). Compared with the 5% glucose-treated (vehicle) group, mice treated with OXA showed increased paw withdrawal frequency (PWF) in response to 0.07-g (Fig. 1, *B* and *C*) and 0.4-g von Frey filaments (Fig. 1, *D* and *E*), as well as decreased paw withdrawal latency (PWL) to cold stimulus (Fig. 1*F*), indicating the presence of mechanical allodynia and cold hyperalgesia. These findings demonstrate that sustained administration of OXA induces peripheral neuropathic pain in mice, largely replicating the clinical manifestations of chemotherapy.

TET1 has been shown to play a crucial role in neuropathic pain (14). Recent evidence demonstrated that TET1 was involved in OXA-induced neuropathic pain by mediating the demethylation of the *SOX10* promoter in the spinal cord of rats (16). In the current study, we investigated the role of DRG TET1 in OXA-induced neuropathic pain. Our data showed that OXA significantly downregulated the TET1 protein (Fig. 1, *G* and *H*, *p* = 0.0203) and mRNA (Fig. 1*I*, *p* = 0.018), by 52.83% and 31.85%, respectively. We also examined the expression of *Sox10* mRNA in DRG after OXA treatment. Interestingly, we found no change in *Sox10* mRNA in OXA-treated DRG (Supporting information Fig. S1). To investigate TET1 distribution in pain-related neurons in the DRG (23), we employed neuron (NeuN) antibodies to label neurons, neurofilament 200 (NF200) for large neurons (24), calcitonin gene-related peptide (CGRP) for medium and small diameter peptidergic neurons (24, 25), isolectin B4 (IB4) for small diameter nonpeptidergic neurons (24, 26), and glutamine synthetase (GS) for satellite cells (27). Dual-labeling experiments showed that TET1 co-existed with NeuN-, NF200-, CGRP-, and IB4-positive cells but was not detected in GS-positive cells (Fig. 1, *J*–*P*).

### Knocking down TET1 in the DRG induces pain hypersensitivity

**Figure 2:**
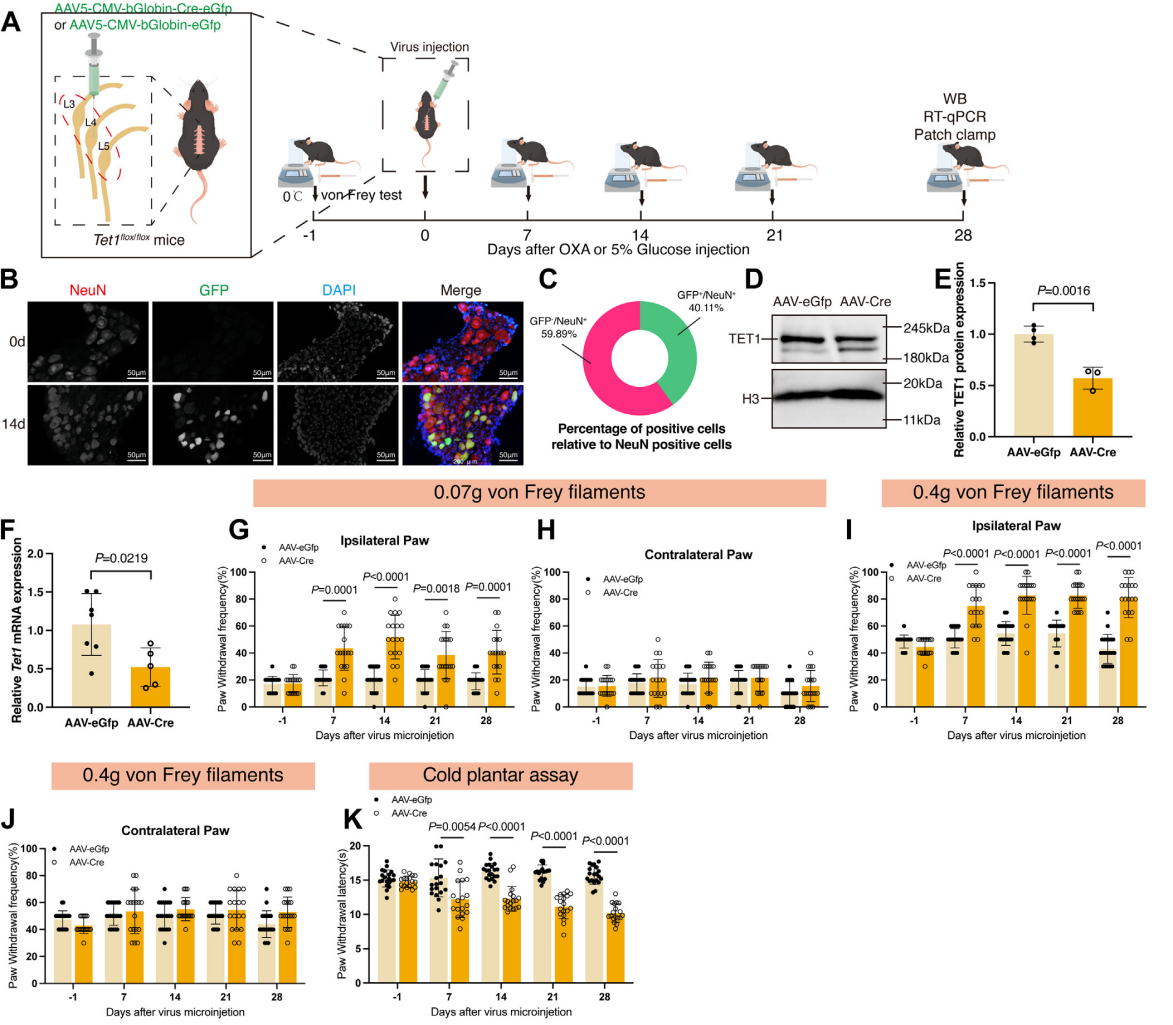
**Specific knockdown of TET1 mimicked OXA-induced pain hypersensitivity.***A*, schematic of the TET1 knockdown and behavioral test strategies. *B*, expression of GFP (*green*), NeuN (*red*), and DAPI (*blue*) in DRG at days 0 and 14 after injection of AAV-eGfp (*n* = 10). Scale bar represents 50 μm. *C*, the proportion of GFP-positive neurons in the DRG was quantified, showing that 40.11% of neurons expressed GFP protein (*n* = 10). *D*–*F*, the DRG was evaluated on day 28. Protein (*D* and *E*) and mRNA (*F*) expressions of TET1 in the AAV-Cre group were downregulated after virus microinjection (unpaired *t* test. Each sample contained at least six DRG from two mice. *n* = 4 in the AAV-eGfp group and *n* = 3 in the AAV-Cre group in D–E. *n* = 7 in the AAV-eGfp group and *n* = 5 in the AAV-Cre group in F). *G*–*J*, in the AAV-Cre group, the PWF in the ipsilateral hind paw increased in response to 0.07 g (*G*) and 0.4 g (*I*) von Frey filament stimulation starting 7 days postinjection, compared to the AAV-eGfp group. However, there was no change in the contralateral hind paw (*H*, *J*) compared to the AAV-eGfp group (two-way ANOVA. *n* = 20 in AAV-eGfp group and n = 18 in AAV-Cre group). *K*, TET1 knockdown induced cold hyperalgesia (two-way ANOVA. *n* = 20 in AAV-eGfp group and *n* = 18 in AAV-Cre group). AAV, Adeno-associated virus; DRG, dorsal root ganglia; GFP, green fluorescent protein; PWF, paw withdrawal frequency; PWL, paw withdrawal latency.

In this study, we addressed the question: Is the decreased TET1 in DRG sufficient for developing OXA-induced pain hypersensitivity? We microinjected the AAV5-CMV-bGlobin-Cre-eGfp (AAV-Cre) virus into the left L3–L5 DRG of *Tet1*^*flox/flox*^ mice. The AAV5-CMV-bGlobin-eGfp (AAV-eGfp) virus was used as a control (Fig. 2*A*). After observing NeuN-positive and expressed GFP-positive cells in the DRG postvirus injection (Fig. 2*B*), we determined that 40.11% of DRG neurons were successfully infected with the adeno-associated virus (AAV) (Fig. 2*C*). As expected, a marked reduction in the levels of TET1 protein (Fig. 2, *D* and *E*, *p* = 0.0016) and mRNA (Fig. 2*F*, *p* = 0.0219) were detected on day 28 postmicroinjection of AAV-Cre compared to the AAV-eGfp–treated groups. Behavioral testing showed that AAV-Cre microinjection produced marked mechanical allodynia and cold hyperalgesia in the ipsilateral paw (Fig. 2, *G*, *I* and *K*). This was demonstrated by the increase in PWF and reduction in PWL, respectively, from days 7 to 28 postOXA. No changes in the basal or contralateral paw withdrawal responses were detected after viral injections (Fig. 2, *G*–*K*).

### TET1 overexpression relieved OXA-induced pain

**Figure 3:**
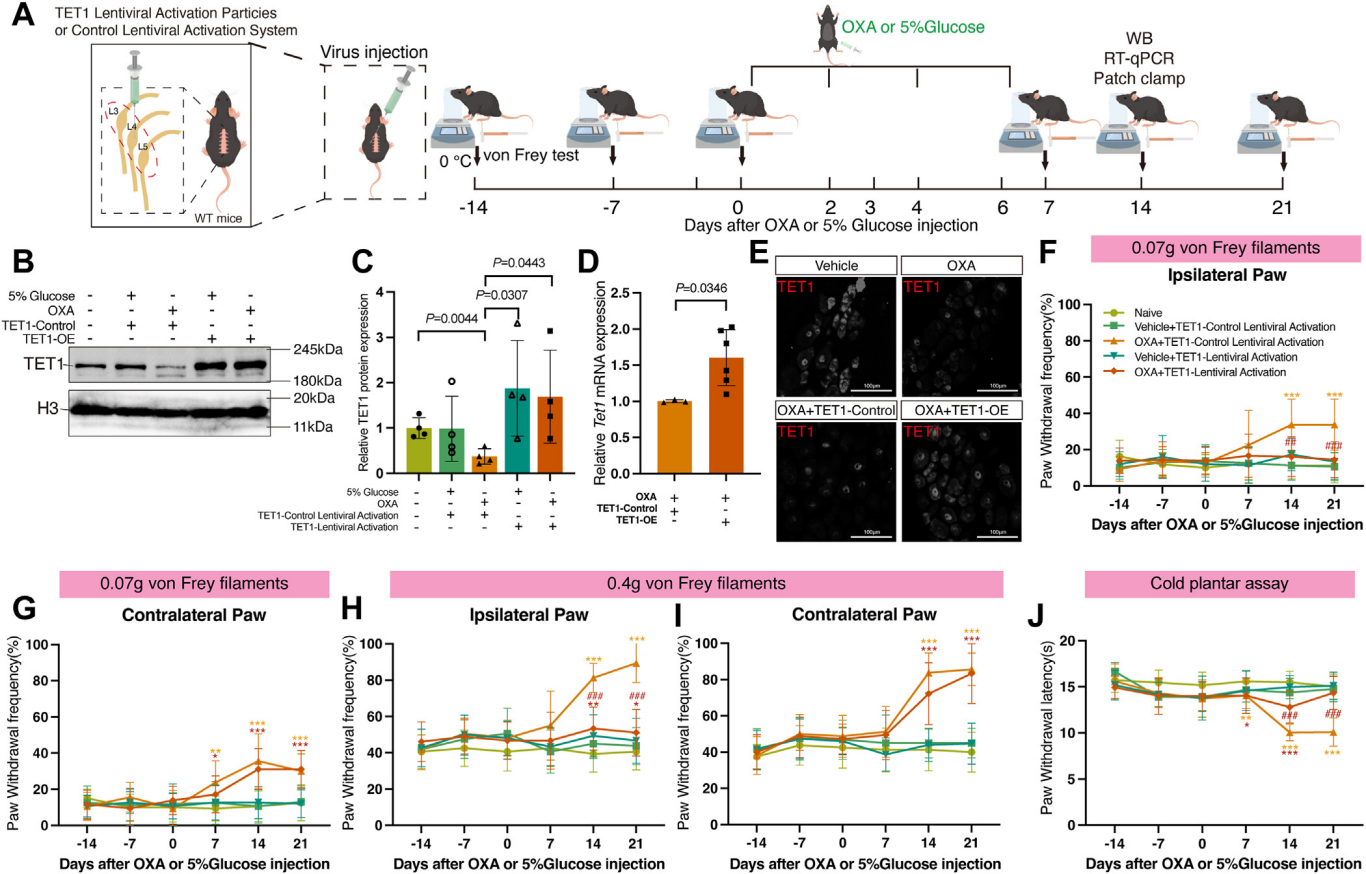
**TET1 overexpression alleviated OXA-induced mechanical allodynia and cold hyperalgesia.***A*, the virus was microinjected into DRG 14 days before the experiment. Then, we injected 5% glucose/OXA intraperitoneally and detected DRG 14 days later. *B* and *C*, on day 14 after injection of OXA or 5% glucose, the expression of TET1 protein increased in the OXA+TET1-OE group compared to the OXA+TET1-Control group (unpaired *t* test. Each sample contained at least six DRG from two mice, *n* = 4 in each group). *D*, the expression of *Tet1* mRNA in the OXA+TET1-OE group was upregulated compared to the OXA+TET1-Control group (unpaired *t* test. Each sample contained at least six DRG from two mice. *n* = 3 in OXA+TET1-Control group and *n* = 6 in OXA+TET1-OE group). *E*, immunofluorescent labeling of TET1-positive cells in vehicle, OXA, OXA+TET1-Control, and OXA+TET1-OE groups of DRG (*n* = 3). Scale bar represents 50 μm. *F*–*I*, upon stimulation with a 0.07 g/0.4 g von Frey filament, both paws of the OXA+TET1-Control group and the contralateral paw of the OXA+TET1-OE group showed mechanical allodynia compared to the Naive group. However, the ipsilateral paw of the OXA+TET1-OE group demonstrated a higher mechanical pain threshold than the OXA+TET1-Control group (two-way ANOVA. *n* = 15 in Veh+TET1-OE group, *n* = 18 in OXA+TET1-OE group, and *n* = 16 for others). *J*, relative to the Naive group, mice in both the OXA+TET1-Control and OXA+TET1-OE groups displayed cold hyperalgesia on days 7 and 14 postinjection. However, by day 14 postinjection, cold hyperalgesia was alleviated in the OXA+TET1-OE group compared to the OXA+TET1-Control group. By day 21 postinjection, the PWL in the OXA+TET1-OE group equaled that of the Naive group (two-way ANOVA. *n* = 15 in Veh+TET1-OE group, *n* = 18 in OXA+TET1-OE group, and *n* = 16 for others). DRG, dorsal root ganglia; OXA, oxaliplatin; PWF, paw withdrawal frequency; PWL, paw withdrawal latency; TET1-OE, TET1-Lentiviral Activation Particles; TET1-Control, TET1-Control Lentiviral Activation System; Veh, vehicle.

To further validate the role of TET1 in neuropathic pain, we upregulated TET1 in the DRG to assess its impact on the hyperalgesia induced by OXA. Fourteen days prior to administering OXA or 5% glucose, we injected TET1 Lentiviral Activation Particles (TET1-OE) into the left L3-L5 DRG of mice to induce TET1 overexpression. The control group received injections of the Control Lentiviral Activation System (TET1-Control) (Fig. 3*A*). The effect of TET1 lentivirus administration was demonstrated by its ability to overexpress the TET1 protein (Fig. 3, *B* and *C*, *p* = 0.0443 *vs*. OXA+TET1-Control) and mRNA (Fig. 3*D*, *p* = 0.0346 *vs*. OXA+TET1-Control). Furthermore, we utilized immunofluorescence to label TET1-positive cells in DRG, and the results were consistent with the findings mentioned above (Fig. 3*E*). Moreover, microinjection of TET1 Lentiviral Activation Particles—but not control particles—ameliorated OXA-induced mechanical allodynia (Fig. 3, *F*–*I*) and cold hyperalgesia (Fig. 3*J*).

### TET1 can reduce neuronal excitability by prolonging the action potential rise time

**Figure 4:**
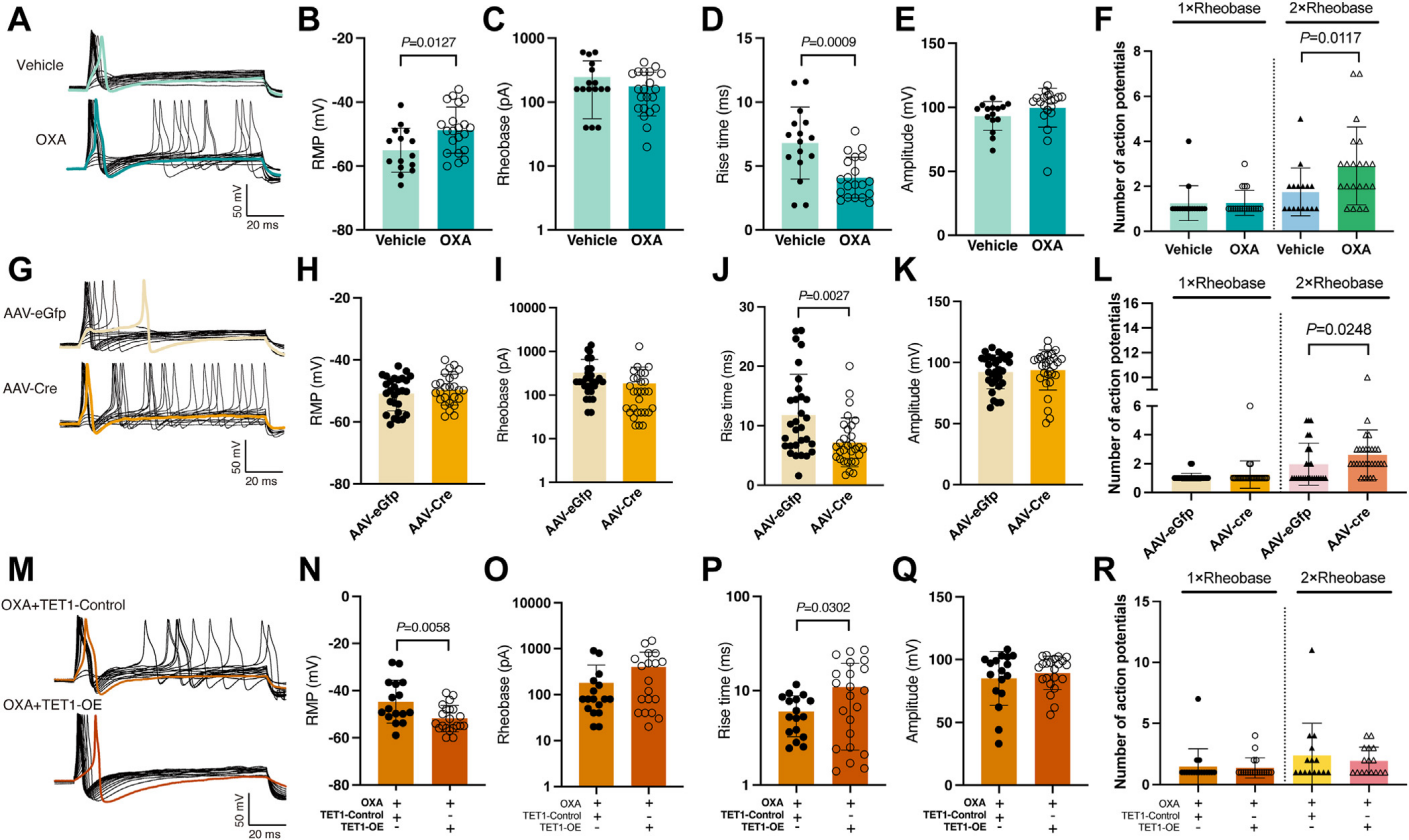
**TET1 inhibits the OXA-induced increase in action potentials in DRG neurons.***A*, representative action potential traces of DRG neurons. *B*–*E*, the RMP (*B*) of DRG neurons in the OXA group was higher, and the rise time (*D*) was shorter than the vehicle group. However, rheobase (*C*) and amplitude (*E*) were similar to those in the vehicle group (unpaired *t* test. *n* = 15–16 in Veh group and *n* = 20–21 in OXA group). *F*, the spike number at twice the rheobase in DRG neurons of the OXA group was increased compared to the vehicle group (unpaired Mann Whitney test. *n* = 16 in Veh group and *n* = 19–21 in OXA group). *G*, representative action potential traces of DRG neurons. *H*, the RMP of DRG neurons in the AAV-Cre group was similar to that in the AAV-eGfp group (unpaired *t* test. *n* = 28 in the AAV-eGfp group and *n* = 26 in the AAV-Cre group). *I*–*K*, the rise time (*J*) of DRG neurons in the AAV-Cre group was shorter than in the AAV-eGfp group, while rheobase (*I*) and amplitude (*K*) were similar to those in the AAV-eGfp group (unpaired *t* test. *n* = 29 for I, *n* = 30 for J, and *n* = 30 for (*K*). *L*, the spike number at twice the rheobase in DRG neurons of the AAV-Cre group was increased compared to the AAV-eGfp group (unpaired Mann Whitney test. *n* = 26–28 in AAV-eGfp group and *n* = 29 in AAV-Cre group). *M*, representative action potential traces of DRG neurons. *N*, the RMP was significantly decreased in the OXA+TET1-OE group compared to OXA+TET1-Control group (unpaired *t* test. *n* = 16 in OXA+TET1-Control group and *n* = 21 in OXA+TET1-OE group). *O*–*Q*, the rise time (*P*) was longer in the OXA+TET1-OE group compared to the OXA+TET1-Control group, while rheobase (*O*) and amplitude (*Q*) were similar to those in the OXA+TET1-Control group (unpaired *t* test. *n* = 17–22). *R*, there was no difference in the spike number at twice the rheobase between DRG neurons of the OXA+TET1-OE group and the OXA+TET1-Control group (unpaired Mann Whitney test. *n* = 15–17 in OXA+TET1-Control group and *n* = 16–19 in OXA+TET1-OE group). AAV, Adeno-associated virus; DRG, dorsal root ganglia; GFP, green fluorescent protein; OXA, oxaliplatin; RMP, resting membrane potential; TET1-Control, TET1-Control Lentiviral Activation System; TET1-OE, TET1-Lentiviral Activation Particles; Veh, vehicle.

To confirm the effect of OXA treatment on the DRG neuronal activity, we evaluated the excitability of small- and medium-sized (Supporting information Fig. S2) nociceptors using whole-cell current-clamp recording (Fig. 4*A*). The results showed that the resting membrane potential (RMP) was significantly depolarized by OXA treatment compared to vehicle treatment (Fig. 4*B*, *p* = 0.0127). Although the rheobase remained unchanged in the OXA group compared to the vehicle group (Fig. 4*C*), the spike number at twice the rheobase significantly increased in the OXA group (Fig. 4*F*, *p* = 0.0117). In addition, we analyzed the rise time of action potentials in each group and found that the rise time was significantly shorter in the OXA group than in the vehicle group (Fig. 4*D*, *p* = 0.0009). No significant differences were observed in the action potential amplitude between these two groups (Fig. 4*E*). These results suggest that OXA treatment induces neuronal hyper-excitability in DRG, which may result from the significantly increased depolarizing ability.

To examine TET1’s regulatory impact on neuronal excitability (Fig. 4*G*), we knocked down TET1 in *Tet1*^*flox/flox*^ mouse DRG, resulting in increased spike numbers (Fig. 4*L*, *p* = 0.0248). Despite this, no differences were found in RMP (Fig. 4*H*), rheobase (Fig. 4*I*), or action potential amplitude (Fig. 4*K*) between the AAV-Cre and AAV-eGfp groups. Additionally, similar to the OXA group (Fig. 4*D*), the rise time of action potentials was significantly shortened by TET1 knockdown (Fig. 4*J*, *p* = 0.0027).

Meanwhile, no difference in action potential counts was found in DRG neurons between the OXA+TET1-OE and OXA+TET1-Control groups (Fig. 4, *M* and *R*), while the RMP was significantly lowered in the OXA+TET1-OE group compared to the OXA+TET1-Control group (Fig. 4*N*, *p* = 0.0058). Furthermore, there were no significant differences in rheobase between the OXA+TET1-OE and OXA+TET1-Control groups (Fig. 4*O*). Notably, a higher proportion of neurons failed to fire in response to stimulation in the OXA+TET1-OE group (9 out of 30 neurons) compared to the OXA+TET1-Control group (2 out of 18 neurons). Additionally, TET1 overexpression reversed the shortened action potential rise time induced by OXA treatment (Fig. 4*P*, *p* = 0.0302), while the amplitude was not affected (Fig. 4*Q*). Further, separate statistical analyses of small- (Supporting information Fig. S3) and medium-sized (Supporting information Fig. S4) neurons confirmed that their outcomes were consistent with the overall results.

### Administration of OXA results in an increase in Nav1.6 expression

**Figure 5:**
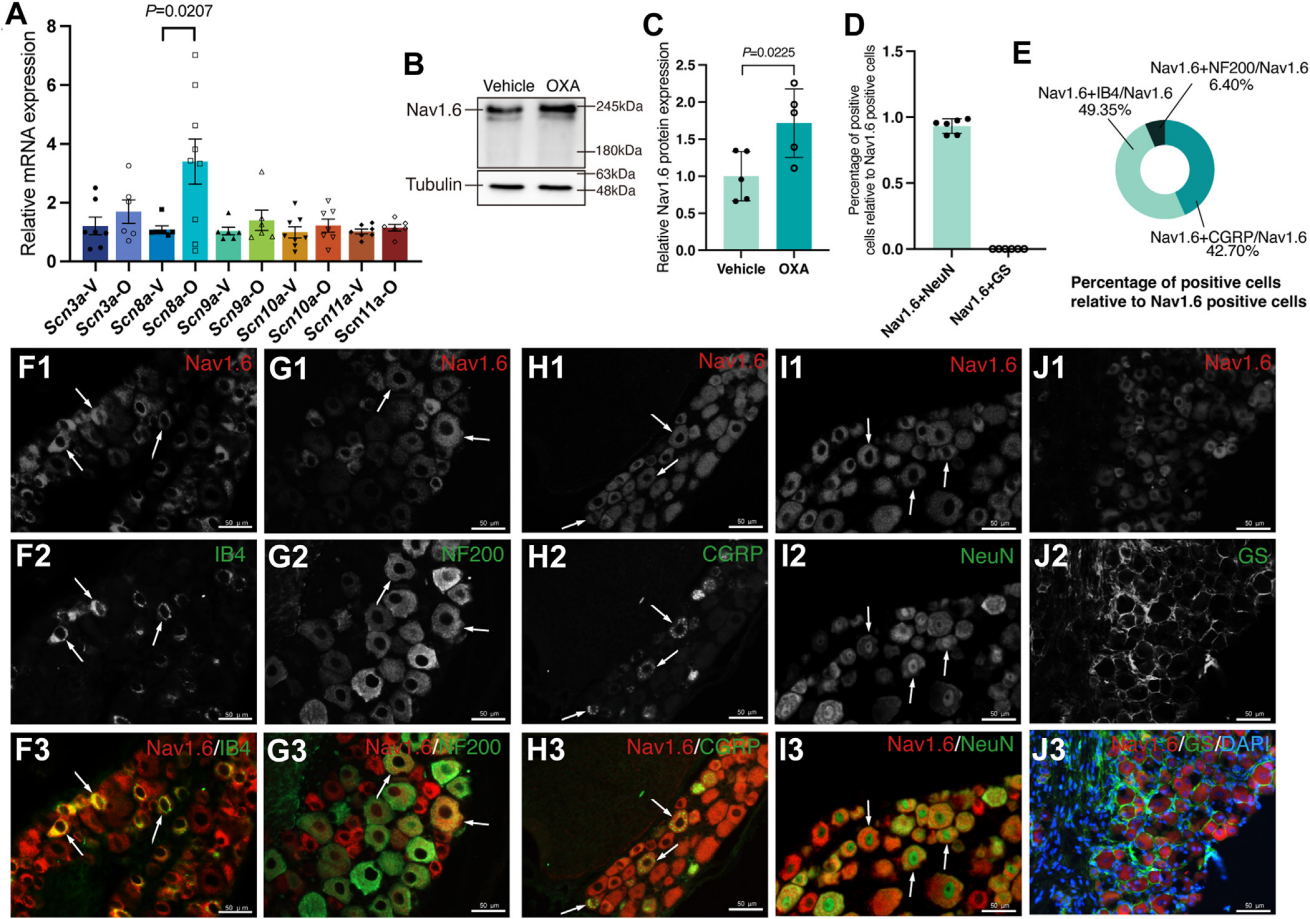
**OXA upregulated Nav1.6 in DRG neurons.***A*, fourteen days post-OXA injection, while *Scn3a*, *Scn9a*, *Scn10a*, and *Scn11a* mRNA levels remained unchanged, only *Scn8a* mRNA was significantly upregulated in the OXA group compared to the Vehicle group (unpaired *t* test. *Scn3a n* = 6, *Scn8a n* = 7, *Scn9a n* = 6, *Scn10a n* = 7, and *Scn11a n* = 6). *B* and *C*, expression of Nav1.6 protein was increased after OXA injection on day 14 (unpaired *t* test. *n* = 5). *D*, percentage of Nav1.6+NeuN- and Nav1.6+GS-positive cells relative to Nav1.6-positive cells (unpaired *t* test. *n* = 6). *E*, percentage of Nav1.6+NF200-, Nav1.6+IB4-, and Nav1.6+CGRP-positive cells relative to Nav1.6-positive cells (*n* = 5 in Nav1.6+CGRP/Nav1.6, *n* = 4 in Nav1.6+IB4/Nav1.6, and *n* = 5 in Nav1.6+NF200/Nav1.6). *F*–*I*, cells (arrows) were labeled for Nav1.6 (*red*), IB4 (*green*), NF200 (*green*), CGRP (*green*), and NeuN (*green*) in WT mice (*n* = 5). Scale bar represents 50 μm. *J*, immunofluorescent Nav1.6 (*red*), GS (*green*), and DAPI (*blue*) triple-labeling of DRG cells (*arrows*) in WT mice (*n* = 5). Scale bar represents 50 μm. CGRP, calcitonin gene-related peptide; GS, glutamine synthetase; IB4, isolectin B4; NeuN, neuron; NF200, neurofilament 200; WT, wild type.

Previous studies have revealed that fast-activated voltage-sensitive Na^+^ channels mainly contribute to depolarization (28). We performed reverse transcription quantitative polymerase chain reaction (RT-qPCR) analysis on day 14 after the first OXA injection. Compared with the vehicle group, OXA significantly upregulated the sodium voltage-gated channel alpha subunit 8 (*Scn8a*) *mRNA* expression (*p* = 0.0207) in DRG (Fig. 5*A*). Similarly, the expression of Nav1.6 protein was also increased (Fig. 5, *B* and *C*, *p* = 0.0225). However, the expression of *Scn3a, Scn9a, Scn10a, and Scn11a* mRNA remains unchanged after OXA treatment (Fig. 5*A*). Additionally, double-labeled immunofluorescence staining was performed to determine the distribution and localization of Nav1.6 (Fig. 5, *D*–*J*). Nav1.6 was expressed in neurons (Fig. 5, *D*, *I* and *J*), primarily in small- and medium-sized DRG neurons (Fig. 5, *E*–*H*). The distribution pattern of Nav1.6 was similar to that of TET1 in mice (Fig. 1, *J*–*P*), which provides physical and spatial evidence for interactions between them.

### TET1 downregulates Nav1.6 protein expression and decreases Nav1.6 current

**Figure 6:**
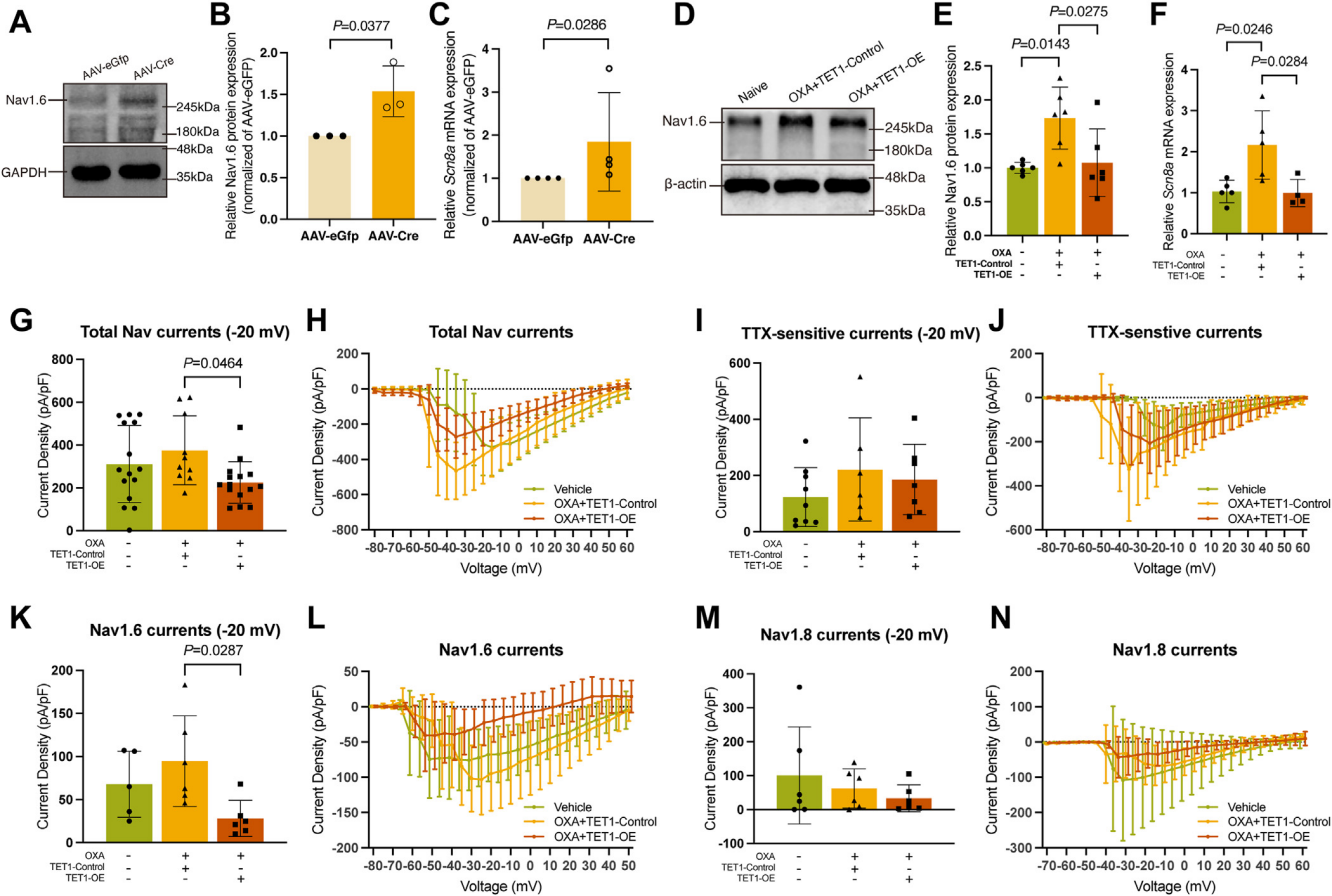
**Overexpression of TET1 decreases Nav1.6 protein expression and Nav1.6 current.***A*–*C*, expressions of Nav1.6 protein (*A* and *B*) and mRNA (*C*) were increased after TET1 knockdown on day 28 (unpaired *t* test. Each sample contained at least six DRG from two mice. *n* = 3 in *A*–*B* and *n* = 4 in (*C*). *D* and *E*, TET1 overexpression blocked the increase in Nav1.6 caused by OXA (one-way ANOVA. Each sample contained at least six DRG from two mice. *n* = 6 in each group). *F*, *Scn8a* mRNA levels increased in the OXA+TET1-Control group relative to the vehicle group but decreased in the OXA+TET1-OE group compared to the OXA+TET1-Control group (one-way ANOVA. Each sample contained at least six DRG from two mice. *n* = 5 in vehicle group, *n* = 5 in OXA+TET1-Control group, and *n* = 4 in OXA+TET1-OE group). *G* and *H*, the total Nav currents density (−20 mV) was lower in the OXA+TET1-OE group than the OXA+TET1-Control group (one-way ANOVA. *n* = 15 in Veh and OXA+TET1-OE groups, and *n* = 10 in OXA+TET1-Control group). *I* and *J*, there was no difference in TTX-sensitive currents density (−20 mV) among the three groups (one-way ANOVA. *n* = 9 in Veh, *n* = 6 in OXA+TET1-Control groups, and *n* = 7 in OXA+TET1-OE group). *K* and *L*, the Nav1.6 currents density (−20 mV) was lower in the OXA+TET1-OE group than in the OXA+TET1-Control group (one-way ANOVA. *n* = 5 in Veh group, and *n* = 6 in OXA+TET1-OE and OXA+TET1-Control groups). *M* and *N*, there was no difference in Nav1.8 currents density (−20 mV) among the three groups (one-way ANOVA. *n* = 6). DRG, dorsal root ganglia; GFP, green fluorescent protein; OXA, oxaliplatin; TET1-Control, TET1-Control Lentiviral Activation System; TET1-OE, TET1-Lentiviral Activation Particles.

To confirm if Nav1.6 acts downstream signaling protein of TET1, we downregulated TET1 by microinjection of AAV-Cre in *Tet1*^*flox/flox*^ mice. As expected, microinjection of AAV-Cre, but not AAV-Gfp, induced the increase in Nav1.6 protein (Fig. 6, *A* and *B*, *p* = 0.0377) and *Scn8a* mRNA (Fig. 6*C*, *p* = 0.0286). Microinjection of TET1 Lentiviral Activation Particles, unlike control particles, reversed the OXA-induced increases in both Nav1.6 protein (Fig. 6, *D* and *E*, *p* = 0.0275 vs. OXA+TET1-Control) and *Scn8a* mRNA levels (Fig. 6*F*, *p* = 0.0284 vs. OXA+TET1-Control).

Nav currents were recorded in small- and medium-sized DRG neurons from the vehicle, OXA+TET1-Control, and OXA+TET1-OE groups (Supporting information Fig. S5*A*). A low concentration of TTX (100 nM) was used to block TTX-s currents, including Nav1.6 currents. Considering potential voltage-clamp errors may be provided by large Nav currents, the current density at −20 mV was measured and used for comparisons of Nav currents between groups. The result showed that TET1 overexpression significantly reduced the total Nav currents amplitudes compared to controls, aligning with reductions in Nav1.6 protein levels (Supporting information Figs. S5*B*, 6, *G* and *H*, *p* = 0.0464 *vs*. OXA+TET1-Control). No significant difference in TTX-s currents was observed between the OXA+TET1-Control group and the OXA+TET1-OE group (Fig. 6*I*). However, OXA trended to increase TTX-s currents in the OXA+TET1-Control group compared to the vehicle group (Fig. 6, *I* and *J*). In addition, a trend of decrease without significant difference in TTX-resistant (TTX-r) currents was also found in the OXA+TET1-OE group when compared with the OXA+TET1-Control group, indicating a potential influence of TET1 on the expression or function of TTX-r Nav channels (Supporting Information Fig. S5, *C* and *D*).

To elucidate TET1’s regulatory effects on Nav1.6 more specifically, we blocked Nav1.6 in DRG neurons using a novel Nav1.6 selective blocker zandatrigine (NBI-921352) to selectively isolate the Nav1.6 currents. The results showed a trend toward increased Nav1.6 currents (−20 mV) in the OXA+TET1-Control group compared to the vehicle group, whereas TET1 overexpression markedly reduced these currents (Fig. 6, *K* and *L*, *p* = 0.0287 *vs*. OXA+TET1-Control). Considering the potential role of TET1 on TTX-r Nav channels, the currents of Nav1.8 (−20 mV) were assessed additionally. However, neither OXA treatment nor TET1 overexpression influenced these currents (Fig. 6, *M* and *N*). These results indicate that TET1 overexpression reduces Nav1.6 currents in DRG neurons of OXA-treated mice and does not influence Nav1.8 currents.

### TET1 binds to and demethylates the *Mir30b* promoter in DRG neurons

**Figure 7:**
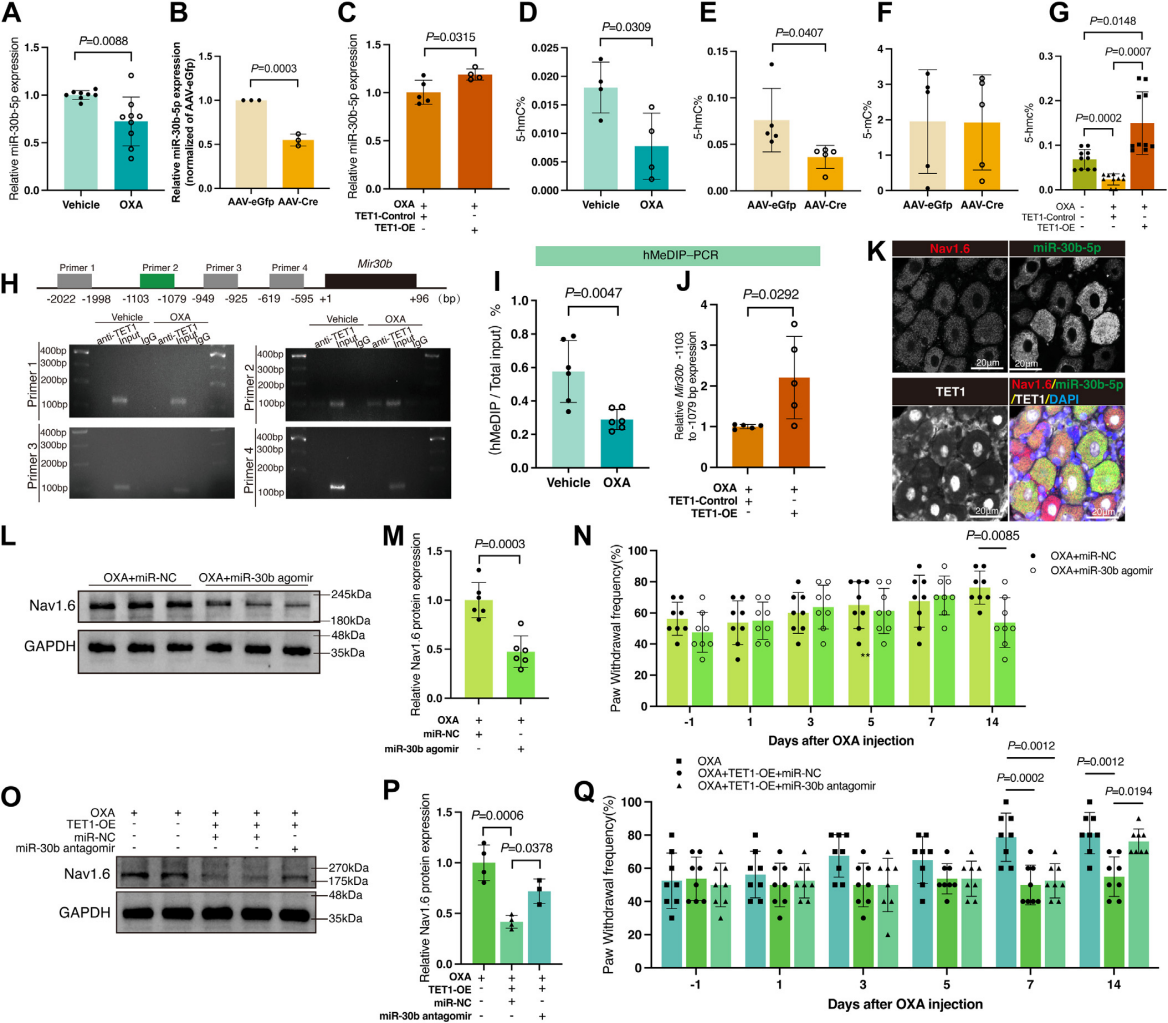
**TET1 modulates Nav1.6 and pain *via* miR-30b.***A*, miR-30b-5p expression in the DRG of the OXA group was decreased compared to the vehicle group (unpaired *t* test. *n* = 8 in Veh group and *n* = 9 in OXA group). *B*, miR-30b-5p expression in the DRG of AAV-Cre group mice was lower than the AAV-eGfp group (unpaired *t* test. Each sample contained at least six DRG from two mice. *n* = 3). *C*, fourteen days after DRG microinjection, TET1 upregulation could alleviate the decrease in miR-30b-5p caused by OXA injection (unpaired *t* test. Each sample contained at least six DRG from two mice. *n* = 5 in OXA+TET1-Control group and *n* = 4 in OXA+TET1-OE group). *D*, genomic DNA hydroxymethylation levels in the DRG decreased after OXA injection (unpaired *t* test. *n* = 4). *E* and *F*, after TET1 knockdown, DNA demethylation levels (*E*) in the DRG of AAV-Cre group mice decreased compared to the AAV-eGfp group; however, DNA methylation levels (*F*) remained consistent between the two groups (unpaired *t* test. Each sample contained at least six DRG from two mice. *n* = 5 in *E* and *F*). *G*, the whole genome DNA hydroxymethylation level increased after TET1 overexpression in the OXA+TET1-OE group compared to the OXA+TET1-Control group (one-way ANOVA. Each sample contained at least six DRG from two mice. *n* = 10). *H*, detection of possible TET1-binding sites in the *Mir30b* promoter region by CHIP (each sample contained at least 30 DRG from five mice, *n* = 2). *I*, demethylated DNA levels at the *Mir30b* promoter in the DRG decreased following OXA injection (unpaired *t* test. *n* = 6). *J*, following TET1 overexpression, demethylation levels in the *Mir30b* promoter region from −1103 to −1079 bp in mouse DRG were enhanced (unpaired *t* test. Each sample contained six DRG from two mice, *n* = 5). *K*, *in situ* hybridization to label miR-30b-5p (*green*) positive cells in DRG. Simultaneously, immunofluorescent labeling was employed to mark Nav1.6 (*red*) and TET1 (*white*) positive cells in DRG (*n* = 5). Scale bar represents 20 μm. *L* and *M*, intrathecal injection of miR-30b agomir reduces the OXA-induced elevation of Nav1.6 protein levels (unpaired *t* test. Each sample contained six DRG from two mice, *n* = 5). *N*, the PWF of the ipsilateral paw in response to 0.4 g von Frey filament stimulation was lower in the OXA+miR-30b agomir group than the OXA+miR-NC group (two-way ANOVA. *n* = 8). *O* and *P*, overexpressing TET1 in OXA-treated mice reduced Nav1.6 protein levels, while intrathecal injection of miR-30b antagomir reversed this effect (one-way ANOVA. Each sample contained six DRG from two mice, *n* = 4 OXA/OXA+TET1-OE+miR-NC, *n* = 3 OXA+TET1-OE+miR-30b antagomir). *Q*, on days 7 and 14 after OXA injection, the PWF of the ipsilateral paw in the OXA+TET1-OE+miR-NC group in response to 0.4 g von Frey filament stimulation was lower than that in the OXA group. However, on day 14, the PWF in the OXA+TET1-OE+miR-30b antagomir group was higher than that in the OXA+TET1-OE+miR-NC group (two-way ANOVA. *n* = 8). AAV, Adeno-associated virus; DRG, dorsal root ganglia; GFP, green fluorescent protein; OXA, oxaliplatin; TET1-OE, TET1-Lentiviral Activation Particles; TET1-Control, TET1-Control Lentiviral Activation System; Veh, vehicle.

TET1, a DNA demethylase, catalyzes the oxidation of 5 mC to 5hmC, regulating gene expression. Despite TET1 knockdown reducing downstream protein, Nav1.6 expression increases. This suggests the involvement of additional molecules in the TET1/Nav1.6 signaling pathway. Our previous research demonstrated that OXA administration decreased the miR-30b-5p expression while increasing the Nav1.6 expression in rat DRG (29). This study revealed that miR-30b-5p was observably downregulated in OXA-treated DRG (Fig. 7*A*, *p* = 0.0088), which is consistent with our previous finding in rats (29). These data suggest the conserved expression pattern of miR-30b-5p during pain development in rats and mice. Knocking down TET1 reduced the miR-30b-5p level (Fig. 7*B*, *p* = 0.0003) while increasing TET1 attenuated the downregulation of miR-30b-5p in DRG (Fig. 7*C*, *p* = 0.0315). Moreover, the effects of OXA on genome-wide demethylation were examined. As shown in Figure 7*D*, compared with the vehicle group, the level of 5-hmc% was markedly reduced after OXA treatment (Fig. 7*D*, *p* = 0.0309), further demonstrating the involvement of DNA demethylation in OXA-related pain. The level of DNA hydroxymethylation was also significantly reduced by AAV-Cre microinjection (Fig. 7*E*, *p* = 0.0407), but the level of DNA methylation did not change (Fig. 7*F*). Overexpression of TET1 blocks the decrease in hydroxymethylation in DRG 14 days post-OXA treatment (Fig. 7*G*, *p* = 0.0007 *vs.* OXA+TET1-Control).

To assess TET1's binding to the *Mir30b* promoter and its regulation of miR-30b-5p, we designed four primer pairs targeting potential methylation sites (−2022 bp to −1998 bp, −1103 bp to −1079 bp, −949 bp to −925 bp, and −619 bp to −595 bp) for chromatin immunoprecipitation (ChIP) analysis from *Mir30b* transcription start site (Fig. 7*H*). Compared to the vehicle group, TET1 binding to the *Mir30b* promoter at −1103 bp to −1079 bp significantly increased in the OXA group (Fig. 7*H*). However, no amplification occurred in the other three regions, indicating TET1’s specific binding to the −1103 to −1079 bp segment of the *Mir30b* promoter in DRG. Subsequently, CpG demethylation at the *Mir30b* promoter was analyzed using the hydroxymethylated DNA immunoprecipitation (hMeDIP) assay. The results indicated that CpG demethylation levels dropped in DRG treated with OXA (Fig. 7*I*, *p* = 0.0047) and a significant increase in the demethylation levels of the promoter region from −1103 bp to −1079 bp of *Mir30b* upon TET1 overexpression (Fig. 7*J*, *p* = 0.0292).

### TET1 downregulates Nav1.6 and alleviates pain through miR-30b

Based on the results, TET1 downregulates Nav1.6 protein and modulates pain. TET1 binds to and increases the demethylation of the *Mir30b* promoter. We hypothesize that *Mir30b* mediates the regulatory effects of TET1 on Nav1.6. We conducted immunofluorescence for TET1 and Nav1.6, along with *in situ* hybridization for miR-30b-5p in mouse DRG neurons, confirming their co-expression in the same neurons (Fig. 7*K*). Seven days post-OXA treatment, mice received intrathecal injections of miR-30b agomir, with controls receiving miR-Negative control (miR-NC). After injection of miRNA, Nav1.6 protein levels in the ipsilateral DRG were reduced in the OXA+miR-30b agomir group compared to the OXA+miR-NC group (Fig. 7, *L* and *M*, *p* = 0.0003). Behavioral assessments revealed a significant reduction in PWF in the ipsilateral hind paw of the OXA+miR-30b agomir group (Fig. 7*N*, *p* = 0.0085), highlighting miR-30b′s role in reducing Nav1.6 expression and alleviating pain.

To determine TET1’s role in regulating Nav1.6 and pain *via* miR-30b, we pre-injected a virus to overexpress TET1 in mouse DRG 14 days before administering OXA. Seven days after the OXA injection, mice received an intrathecal injection of miR-30b antagomir, with controls given equivalent miR-NC. Nav1.6 protein levels were reassessed in the DRG, revealing significantly lower levels in the OXA+TET1-OE+miR-NC group than the OXA group (Fig. 7, *O* and *P*, *p* = 0.0006), aligning with previous findings (Fig. 6, *D* and *E*). However, the OXA+TET1-OE+miR-30b antagomir group showed significantly higher Nav1.6 levels than the OXA+TET1-OE+miR-NC group (Fig. 7, *O* and *P*, *p* = 0.0378). While TET1 overexpression alleviated OXA-induced mechanical allodynia, subsequent miR-30b antagomir injection reversed this relief, reinstating mechanical allodynia (Fig. 7*Q*, *p* = 0.0194). These findings confirm miR-30b′s involvement in TET1-mediated Nav1.6 and pain regulation.

**Figure 8:**
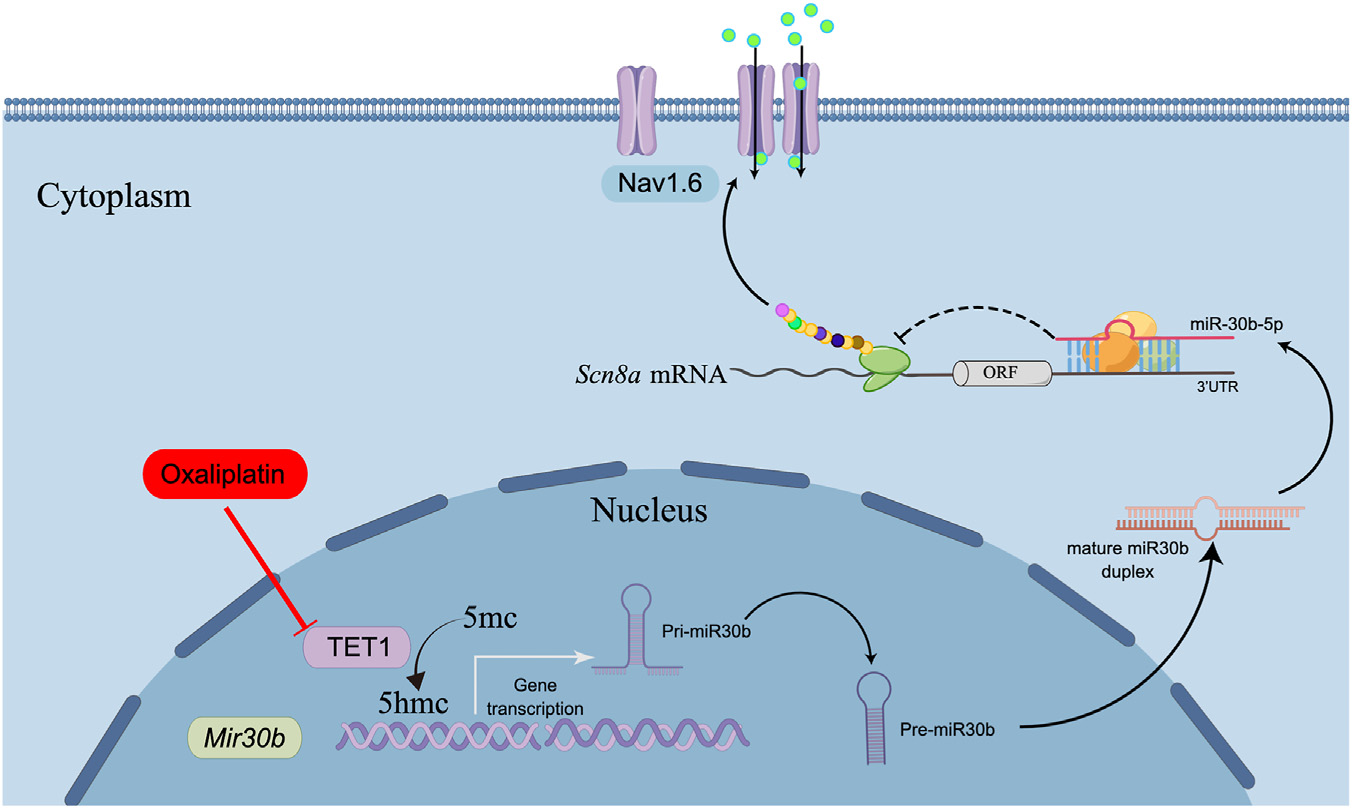
**Proposed mechanism by which TET1 participates in neuropathic pain.** In physiological conditions, TET1 binds to the promoters of *Mir30b* (−1103 bp to −1079) and promotes the expression of miR-30b-5p, which inhibits the expression of Nav1.6. In pathological and chronic pain states, TET1 expression decreases in the nucleus. Lack of TET1 binding to the promoters of *Mir30b* results in a decrease in *Mir30b* transcription and miR-30b-5p expression, leading to an increase in the expression of the Nav1.6 protein. The enhanced Nav currents subsequently facilitate the spike firing of nociceptors. In summary, TET1 participates in oxaliplatin-induced neuropathic pain in mice by regulating miR-30b/Nav1.6 signaling.

Drawing from the data above, it can be inferred that TET1 suppression results in miR-30b-5p reduction, while Nav1.6 augmentation amplifies the neuronal excitability, ultimately culminating in the manifestation of pain (Fig. 8).

## Discussion

To our knowledge, this study provides the first evidence that TET1 regulates miR-30b/Nav1.6 signaling in DRG neurons affected by OXA-induced peripheral neuropathy. Decreased TET1 correlates with lower miR-30b levels, which subsequently upregulates Nav1.6 signaling, leading to neuropathic pain. TET1 overexpression in DRG neurons reverses the reduction of miR-30b, counteracts OXA-induced Nav1.6 upregulation, and alleviates chemotherapy-related pain hypersensitivities.

Recently, epigenetic modification has been extensively studied across fields such as antitumor immunity (30), neurodegeneration, neuroprotection (31), congenital diseases (32), and chronic pain (33). As a demethylase, TET1 is implicated in pathological pain, including neuropathic and inflammatory pain (13, 16, 34). However, TET1's expression pattern and functions are still controversial. For instance, some studies showed TET1 upregulation in the ipsilateral spinal cord dorsal horn in neuropathic and inflammatory pain models, while others found that DRG TET1 overexpression alleviates pain-like behaviors (34, 35). In our study, a decrease in DRG TET1 post-OXA treatment was observed, with its overexpression significantly reducing OXA-induced mechanical and cold allodynia. Our findings align with data showing that DRG TET1 overexpression, targeting μ-opioid receptor and Kv1.2 (13), mitigates neuropathic pain, contrasting with spinal TET1 roles post-OXA or spinal nerve ligation (16). These results highlight the region-specific roles of endogenous TET1 in DRG and spinal dorsal horn neurons in pain genesis.

We analyzed the mRNA expression of pain-related sodium channel genes: *Scn3a* (encoding Nav1.3), *Scn8a* (encoding Nav1.6), *Scn9a* (encoding Nav1.7), *Scn10a* (encoding Nav1.8), and *Scn11a* (encoding Nav1.9). We observed a significant increase in *Scn8a* expression, with stable expression of *Scn3a, Scn9a*, *Scn10a,* or *Scn11a,* highlighting varied expression patterns in neuropathic pain models induced by nerve injury or chemotherapy. Notably, the most significant change was in Nav1.6 expression, correlating with its key role in chemotherapy-induced cold allodynia (36). Additionally, Nav1.6 currents showed a trend toward an increase in the OXA+TET1-Control group relative to the vehicle group, whereas a reduction was found in the OXA+TET1-OE group compared to the OXA+TET1-Control group (Fig. 6*K*). Meanwhile, we also observed a tendency for TTX-r currents to decrease following TET1 overexpression relative to the control group, indicating potential regulation of ion channels beyond Nav1.6 by TET1 (Supporting information Fig. S5*C*). Compared to previous studies, this provides further direct evidence of Nav1.6's involvement in pain induction. However, due to the large diameter and membrane area of DRG neurons, errors due to space clamp issues could be introduced and affect the results of Nav current recording by single patch-clamp recording. Although we selected only small- and medium-sized neurons for whole-cell recording of Nav currents, the potential influence of space clamp issues on our results cannot be completely ruled out. In future studies, employing double patch-clamp recording may more accurately verify these results.

We assessed the impact of TET1 expression on the global 5hmc level through overexpression or knockout experiments. TET1 overexpression substantially restored OXA-impaired 5hmc levels (Fig. 7*G*), while its conditional knockdown significantly reduced 5hmc levels in naïve mice (Fig. 7*E*). Notably, TET1 binds to the methylation-prone *Mir30b* promoter fragment (−1 103 bp to −1 079 bp) (Fig. 7*H*), and its overexpression can reverse OXA-induced demethylation in this region (Fig. 7, *I* and *J*). Further analyses showed that microinjecting TET1-lentiviral vectors into the DRG blocked the OXA-induced increase in Nav1.6 (Fig. 6, *D* and *E*). Microinjection of AAV-Cre into the DRG of *Tet1*^*flox/flox*^ mice elevated Nav1.6 expression (Fig. 6, *A* and *B*), causing pain-like behaviors (Fig. 2, *G*–*K*). Moreover, in OXA-induced pain, decreased TET1 and increased Nav1.6 expression suggest that TET1's regulation of Nav1.6 does not involve demethylation. Subsequently, by using miR-30b agomir and miR-30b antagomir, we demonstrated the regulatory role of miR-30b in Nav1.6 and pain (Fig. 7, *L*–*N*) and confirmed that miR-30b is involved in the regulation of Nav1.6 and pain by TET1 (Fig. 7, *O*–*Q*). These findings confirm TET1's regulation of miR-30b/Nav1.6 and its involvement in OXA-induced neuropathic pain *via* miR-30b/Nav1.6 signaling modulation.

Although our study highlighted TET1/Nav1.6's unique regulatory mechanism in chemotherapy-induced pain, it has several limitations. Firstly, other studies suggested that DNA demethylases and methylases like DNA methyltransferase 3 alpha, which silences Kv1.2 in the spinal dorsal horn, contribute to pain development, including bone cancer pain (37). In addition, TET3 shows differential upregulation postinjury, suggesting its role in neuropathic pain (38). However, we did not detect changes in DNA methyltransferase 3 alpha, TET3, or TET2 expression, warranting further investigation. Secondly, while we outlined TET1 and Nav1.6's regulatory mechanism, it is unknown if TET1 also regulates other ion channels in OXA-induced pain. Thirdly, given the inconsistency of spinal TET1's role in peripheral injury and chemotherapy-induced pain compared to DRG (39, 40), further exploration of post-OXA administration is needed. Fourthly, due to the major role and recording challenges of small- and medium-sized DRG neurons (41), we focused on their excitability and Nav currents, excluding large neurons. However, the potential of TET1 to regulate large neuron excitability and its impact on pain also needs consideration. Lastly, we administered intrathecal injections of miR-30b agomir to OXA-treated mice and miR-30b antagomir to OXA+TET1-OE mice to assess miR-30b′s role in regulating Nav1.6 and to explore TET1’s regulation of Nav1.6 *via* miR-30b (Fig. 7, *L*–*Q*). However, due to technological limitations, we did not directly modify the demethylation levels of the *Mir30b* gene promoter region (−1103 bp to −1079 bp) to confirm these results. Theoretically, increasing demethylation in the *Mir30b* gene promoter should elevate miR-30b; hence, simulating this with intrathecal miR-30b agomir injections is plausible. We conclude that TET1 regulates Nav1.6 and pain *via* miR-30b, hypothesizing that this effect involves TET1’s influence on demethylation in the *Mir30b* gene promoter region (−1103 bp to −1079 bp).

In summary, our study provided new evidence that TET1 upregulated Nav1.6 protein expression by miR-30b, increasing neuronal excitability during pain. Conversely, TET1 overexpression mitigated these effects and alleviated pain. These findings offer valuable insights into the pain pathophysiology and suggest TET1 as a promising target for analgesic drug development.

## Experimental procedures

### Animals

Male C57BL6/J mice, weighing 25 to 30 g, were obtained from the Beijing Vital River. All animals were kept under a 12–12-h light-dark cycle at 24 ± 1 °C and 40 to 60% humidity and had *ad libitum* access to food and water. *Tet1*^*flox/flox*^ mice were gifted by Dr Guoliang Xu (Center for Excellence in Molecular Cell Science, Chinese Academy of Sciences) (42). The procedures for the care and use of animals were approved by the Animal Care and Use Committee of Zhengzhou University and implemented following the guidelines of the International Association for the Study of Pain.

### Drug administration

OXA (JARI Pharmaceutical Co., Ltd) was dissolved in a 5% glucose solution at a concentration of 0.2 mg/ml. To simulate chemotherapy-induced neuropathic pain, 3 mg/kg of OXA was administered intraperitoneally every other day for a total of four treatments. Recombinant AAV5-CMV-bGlobin-Cre-eGfp 1.08E + 13 v.g/ml and AAV5-CMV-bGlobin-eGfp 1.74E + 13 v.g/ml were purchased from GeneChem. AAV (1 μl) was injected into the L3–L5 DRG of *Tet1*^*flox/flox*^ mice on the left side. TET1 Lentiviral Activation Particles (1 μl; 5E + 6 v.g/ml) (Santa Cruz Biotechnology) and the Control Lentiviral Activation System (1 μl; 5E + 6 v.g/ml) (Santa Cruz Biotechnology) were administered to the DRG for 14 days before OXA treatment.

### Behavioral tests

#### Von Frey testing

As previously described, 0.07 g and 0.4 g von Frey filaments (Stoelting, Inc) were used to test the mechanical sensitivity (37). Briefly, the PWF was determined by applying von Frey hairs to the middle of the plantar surface of the hind paws. The frequency of paw withdrawal during the 10 tests was recorded to reflect the sensitivity to mechanical stimuli. The behavioral tests were conducted in a double-blind manner.

#### Cold plate assay

Briefly, animals were placed on the surface of the cold plate (Bioseb) precooled to 0 °C. A built-in timer was started following the placement of animals (43). The operator stopped the timer when animals exhibited paw lifting, flinching, or guarding. The duration between the placement and flinch was recorded as the PWL to noxious cold. The behavioral tests were conducted in a double-blind manner.

### DRG microinjection

Mice were deeply anesthetized with 1.5% isoflurane (RWD, Life Science Co., Ltd). The L3–L5 DRG were surgically exposed under a stereomicroscope. The virus was injected continuously at a rate of 50 nl/min using a 1-μL Hamilton syringe (7001 KH, Hamilton Company). After surgery, muscles and skin were sutured in layers, and the incision site was sterilized with iodophor.

### RNA extraction and quantitative real-time reverse transcription PCR

For the RT-qPCR, bilateral L3–L5 DRG were pooled together to obtain sufficient RNA. Total RNA was extracted using AxyPrep Multisource Total RNA Miniprep Kit (Axygen), according to the manufacturer’s instructions. Reverse transcription was carried out using the Reverse Transcription System (Promega), according to the manufacturer’s instructions. For miRNA RT-qPCR, reverse transcription was conducted using the miRcute Plus miRNA First-Strand cDNA Kit (TianGen), and miRNA amplification was performed using the miRcute Plus miRNA qPCR Kit (TianGen). *Tubulin* and *U6* were used as internal controls for the normalization of mRNA and miRNA, respectively. The relative expression of the target genes was analyzed using the 2^-ΔΔCT^ method. Supporting information Tables S1 and S2 lists the primers used in the study.

### Western blot analysis

To ensure adequate protein quantity, three or six DRG were pooled from either the unilateral or bilateral L3–L5 of mice. The tissues were homogenized using an ice-cold lysis buffer. After electrophoresis, proteins were electroeluted at 250 mA onto polyvinylidene fluoride membranes (Millipore). The membranes were placed in the block buffer for 2 h at room temperature and incubated with the rabbit TET1 antibody (1:1000, GTX124207, GeneTex Inc.), rabbit Nav1.6 antibody (1:200, ASC-009, Alomone Labs), rabbit Histone H3 antibody (1:1000, ab1791, Abcam), rabbit GAPDH antibody (1:1000, ab9485, Abcam), rabbit Tubulin antibody (1:1000, ab6046, Abcam), and rabbit beta-actin antibody (1:1000, ab8224, Abcam) overnight at 4 °C. Then, the membranes were incubated with peroxidase-conjugated secondary antibody for 2 h at room temperature. Finally, enhanced chemiluminescence (Beyotime Biotechnology) was used to detect the immune complexes. Use ImageJ software (version 2.9.0, National Institutes of Health) to convert the image to 8 bit grayscale, calibrate the gel using the “uncalibrated OD” function, and then invert the gel. Next, select the target band and measure to determine the area value.

### Immunofluorescence staining

The L3–5 DRG were collected, postfixed, and dehydrated before being subjected to frozen sectioning to produce 12-μm sections. After washing with 0.01 M PBS, the slides were blocked with 10% goat serum for 2 h and then incubated overnight at 4 °C with the primary antibody, including rabbit anti-TET1 (1:200, GTX124207, GeneTex), mouse anti-NeuN (1:1000, ab104224, Abcam), mouse anti-GS (glutamine synthetase; 1:400, SAB5701312), mouse anti-CGRP (calcitonin gene related peptide; 1:200, ab81887, Abcam), FITC-conjugated IB4 (Isolectin B4, 1:200, L2895, Sigma), mouse anti-NF200 (neurofilament 200; 1:200, N0142, Sigma), and rabbit anti-Nav1.6 (1:200, ASC-009, Alomone Labs). The slides were incubated with cy3-conjugated goat antirabbit and Alexa Fluor 488–conjugated goat antimouse secondary antibodies for 2 h at 37 °C. The stained slides were observed under a Nikon Ni-U fluorescence microscope (Nikon).

### *In situ* hybridization

*In situ* hybridization was performed as described previously (44). On tissue sections, 3% freshly diluted gastric protease was applied for 1-min digestion at room temperature and then washed with 0.5 M PBS washes (3 times, 5 min each). Subsequently, a distilled water rinse was performed once. Add prehybridization solution, incubate at 41 °C for 3 h, add oligonucleotide probes, and incubate overnight at 40 °C. Subsequent washing steps included two 5-min washes with 37 °C 2 × SSC (containing 17.6 g NaCl and 8.8 g C6H5O7Na3·2H2O per 1000 ml distilled water), then washed with 0.5 × SSC for 15 min, and washed with 0.2 × SSC for 15 min. A 37 °C reaction for 30 min with a blocking solution was carried out, followed by the addition of biotinylated mouse antidigoxin and washed four times (5 min each) with 0.5 M PBS. Subsequently, SABC-FITC was allowed to react at 37 °C for 30 min and washed three times (5 min each) with 0.5 M PBS, and observed under a microscope.

### DNA isolation and hMeDIP assay

The DRG were lysed using the DNA lysis buffer (LifeFeng). The genomic DNA was sonicated (JY92-IIDN, Ningbo Scientz Biotechnology Co., Ltd) to produce DNA fragments ranging in size from 100 to 600 bp. DNA fragments containing 5-hydroxymethylcytosine that were isolated from DRG were captured in a high-throughput format using Epiquik hMeDIP Kit (AP1038, EpiGentek). Next, hMeDIP enrichment was performed to isolate DNA fragments containing 5-hmC, followed by quantitative PCR. RT-qPCR was performed using the Quantstudio 3 PCR system (Application Biossystems) with SYBR Green (Thermo Fisher Scientific). The following *Mir30b* promoter primer sequences were used: forward sequence: 5′- ACAGCTTTCAAATACTACTGTGCAT-3′; reverse sequence: 5′- CCAGACTTACTAGGGATGGAAC-3′.

### ChIP assays

ChIP assay was performed using the Chroma Flash High Sensitivity ChIP Kit (Epigentek) according to the manufacturer’s instructions and the methods reported previously (45). Briefly, the L3–L5 DRG were isolated and placed in 1% formaldehyde for 20 min. The DNA was sheared using ultrasound (JY92-IIDN, Ningbo Scientz Biotechnology) and digested with *micrococcus* nuclease. Following the addition of the ChIP dilution buffer, 100 μl of the supernatant was saved as input. The TET1 antibody (GeneTex) was added to 80 μl of the sample for preclearing. The antibody-bead complex was incubated with the chromatin solution overnight at 4 °C. IgG was used as the negative control for immunoprecipitation. The purified DNA was passed through DNA purification columns and eluted in an elution buffer prior to reverse-transcription PCR.

### Hydroxymethylated and methylation DNA quantification (colorimetric)

The genomic levels of DNA hydroxymethylation and methylation were analyzed using the Hydroxymethylated DNA Quantification Kit (Colorimetric) (Epigentek) and MethylFlash Global DNA Methylation 5 mC ELISA Easy Kit (Colorimetric) (Epigentek), respectively. Following the manufacturer’s instructions to ensure the binding of DNA to the assay well, the wells were washed and the captured antibody was added. Next, the wells were washed and the detection antibody and enhancer solution were added. Finally, the color-developing solution was added for color development. The absorbance was measured using an Epoch Microplate Spectrophotometer (BioTek).

### Whole-cell patch clamp recording

Mouse L3-5 DRG were quickly isolated and dissociated with collagenase type I (1 mg/ml) and dispase II (5 mg/ml) for 30 min as previously described (46). Cells were resuspended in complete Neurobasal-A Medium, added to precoated 8 mm coverslips (0.1 mg/ml poly-D-lysine, 20 μl per coverslip), and placed into the incubator (37 °C, 5% CO_2_). All neurons were recorded within 2 to 8 h after resuspension (47). Borosilicate capillary glass pipettes (4–6 MΩ for action potential and 1–2 MΩ for sodium current recording by P-97, Sutter Instruments) were used for whole-cell patch clamp recording. Coverslips with neurons were plated into a thermostatic chamber (Warner, Instruments) mounted on an inverted microscope (Nikon) to maintain the temperature at 35 °C to record the action potential or at 31 °C to record sodium currents. Signals were low-pass-filtered at 2.9 kHz (EPC 10-USB, HEKA) and digitized at 50 kHz using the Patchmaster software (HEKA). The investigator was blinded to the treatment groups during the recording process.

Action potentials were recorded by current-clamp recording. The extracellular solution contained (in mM) the following: 145 NaCl, 3 KCl, 2 MgCl_2_, 2 CaCl_2_, 10 Hepes, and 10 glucose, with pH adjusted to 7.3 to 7.4 using Tris-base. The intracellular pipette solution contained (in mM) the following: 135 KCl, 2 MgCl_2_, 2 Na_2_-ATP, 10 glucose, 10 Hepes, and 10 EGTA, with pH adjusted to 7.2 to 7.3 using KOH and osmolarity adjusted to 300 mOsm using sucrose. The bridge was 100% balanced. The holding current was 0 pA. Three stimulating protocols were used to generate action potentials according to the state of the neuron: 1) 10 to 100 pA in increments of 10 pA and 100 ms; 2) 40 to 600 pA in increments of 40 pA and 100 ms; and 3) 100 to 1500 pA in increments of 100 pA and 100 ms. The RMP was calculated as the mean potential during the 10 ms before the first stimulus pulse in the initial trial. The action potential rheobase was defined as the minimum current required to evoke the first action potential. The action potential amplitude was defined as the range from the RMP to the peak of action potential. The rise time (time to peak) was defined as the time for the membrane potential to increase from the stimulus onset to the peak of the first action potential. The decay time was defined as the time to decrease from the peak membrane potential to a value of 10% of the peak value.

The extracellular solution for sodium currents recording contained (in mM) the following: 70 NaCl, 70 TEA-Cl, 3 KCl, 1 MgCl_2_, 1 CaCl_2_, 10 Hepes, and 10 glucose, with pH adjusted to 7.3 using NaOH. The potassium channels were blocked using TEA-Cl. An additional 200 μM CdCl_2_ was added to the extracellular solution to block calcium channels. The intracellular pipette solution contained (in mM) the following: 140 CsF, 10 NaCl, 2 Mg-ATP, 10 Hepes, and 10 EGTA, with pH adjusted to 7.2 using CsOH. A low concentration of tetrodotoxin (TTX, 100 nM) was used to block about 90% of Nav1.6 currents and some other TTX-s Nav currents. To isolate the Nav1.6 currents more specially, a Nav1.6-special inhibitor zandatrigine (100 nM, HY-147423, MCE) was added to the extracellular solution to inhibit the Nav1.6 channels. The holding voltage was −80 mV. The total, TTX-, and zandatrigine-resistant Nav currents were obtained using a stimulus containing a series of 500-ms pre-pulses at −100 mV, each followed by a test pulse for 90 ms; the current increased in increments of 5 mV from −80 to +60 mV. The TTX- or zandatrigine-sensitive currents were calculated by subtracting the TTX- or zandatrigine-resistant currents from the total Nav currents. The inter-pulse interval was 9 s. The stimulus for Nav1.8 contains a series of 500-ms pre-pulses at −50 mV, each followed by a test pulse for 90 ms; the current increased in increments of 5 mV from −70 to +60 mV; the sweep interval was 1 s. The current density was obtained by dividing the raw currents by the neuron diameters.

### Intrathecal injection

Seven days postinitial OXA injection, mice were anesthetized with 3% isoflurane. Anesthesia was maintained with 1.5% isoflurane as mice were prone to a flat surface. A 15 ml centrifuge tube was positioned under the mouse’s abdomen to arch the spine, enlarging the space between spinous processes. The mouse’s back was shaved and disinfected with 75% alcohol to expose the skin. Using a U-40 insulin needle, 0.5 nmoL of miR-30b agomir or antagomir was drawn and vertically inserted into the spine between the L3-L4 interspinous space. If the needle contacted the spinal nerve, indicated by twitching the mouse’s paws, the solution was slowly injected, followed by careful withdrawal. The injection site was re-disinfected with iodine before placing the mouse in a warm recovery cage. Daily injections of miR-30b agomir or antagomir were administered for five consecutive days. miR-30b agomir sequence (5′ to 3′): (S) UGUAAACAUCCUACACUCAGCU; (AS) CUGAGUGUAGGAUGUUUACAUU. miR-NC sequence (5′ to 3′): (S) UUCUCCGAACGUGUCACGUTT; (AS) ACGUGACACGUUCGGAGAATT. miR-30b antagomir sequence (5′ to 3′): (S) AGCUGAGUGUAGGAUGUUUACA. MicroRNA inhibitor N.C sequence (5′ to 3′): (AS) CAGUACUUUUGUGUAGUACAA.

### Statistical analysis

Data are presented as mean ± SD and analyzed using GraphPad Prism 9. Data points that deviate more than twice the SD from the mean are considered outliers. The normality of distribution for each dataset was assessed before analysis with the same software. Methods to test distribution: D'Agostino-Pearson omnibus normality test; Anderson-Darling test; Shapiro–Wilk normality test; Kolmogorov-Smirnov normality test with Dallal-Wilkinson-Lillie for *p* value. The data were compared using an unpaired *t* test, one-way ANOVA, or two-way ANOVA with repeated measures followed by a Bonferroni post-test. Statistical significance was set at *p* < 0.05. Statistical comparisons between experimental groups for each figure can be found in Supporting Information Table S3.

## Data availability

The datasets generated during and/or analyzed during the current study are available from the corresponding author on reasonable request.

## Ethics approval

The authors’ institution provided ethical approval for the conduct of this study.

## Supporting information

This article contains supporting information.

## Conflict of interest

The authors declare that they have no conflicts of interest with the contents of this article.
