# Supplementary material for: TET1 participates in oxaliplatin-induced neuropathic pain by regulating microRNA-30b/Nav1.6
Source: J Biol Chem. 2025 Jan 27;301(3):108228. doi: 10.1016/j.jbc.2025.108228 (PMC11894311; doi:10.1016/j.jbc.2025.108228)
Supplement: Supporting information [file mmc1.pdf]

TET1 participates in oxaliplatin-induced neuropathic pain by regulating microRNA-30b/Nav1.6

Sen Zhao[1,2], Jing-jing Zhang[1,3,4], Meng-ya Zhang[1], Qing-qing Yang[1,2], Zhi-xiao Li[1], Xiu-hua Ren[1], Song-xue Su[1], Tian-en Si[1], Jian-min Li[1,2], Hui-rui Wu[1], Shi-yue Chen[1], Wei-dong Zang[1,2*], Jing Cao[1,2,3*]

List of the material included.

Table S1……………………………………………………………S-2
Table S2……………………………………………………………S-2
Table S3……………………………………………………………S-2
Figure S1……………………………………………………………S-17
Figure S2……………………………………………………………S-18
Figure S3……………………………………………………………S-19
Figure S4……………………………………………………………S-20
Figure S5……………………………………………………………S-22

Table S1. Primers of *Mir30b* promoter region.

No.		Sequences (5'-3')	Size(bp)
1	Forward	ACATTTGTTGTGTATTATTCTGCCC	112
	Reverse	GCAGATGACGAAAAGGCTGT	
2	Forward	ACAGCTTTCAAATACTACTGTGCAT	97
	Reverse	CCAGACTTACTAGGGATGGAAC	
3	Forward	AATCCAGAGCACTCAAATTATAGGT	100
	Reverse	TAGACACATTTAATTCTTTGGGGTG	
4	Forward	TCCCTGATGAAAGTATTCCAAGAGT	123
	Reverse	GGTAGCAAGGGGTAGCACAA	

Table S2. Primer sequences for RT-qPCR

Sequences (5'-3') of primers used		
**Gene**	**Forward primer**	**Reverse primer**
Scn3a	ATCCTCAACAGTGCACCTCCT	TGTGGCAACGCTGAAGTTCT
Scn8a	AGTAACCCTCCAGAATGGTCCAA	GTCTAACCAGTTCCACGGGTCT
Scn9a	TAGTGGAAATGTGCGATGGA	CTTGAAGCAGCGACAGGTAA
Scn10a	CAGTGTCATTACCCTGGCGT	CGATGACCTTCAGTCCTGGG
Scn11a	ATGTGGGAATGCATGCAGGA	AGAGGTTAAGCACCACGAGC
Tet1	GCAGTGAACCCCGGAAAAC	AGAGCCATTGTAAACCCGTTG
Sox10	CGGACGATGACAAGTTCCCC	GTGAGGGTACTGGTCGGCT
Tubulin	GTGCATCTCCATCCATGTTG	GTGGGTTCCAGGTCTACGAA
miR-30b-5p	GCGCTCGAGTATTGTAGTCTGTT GGTGCC	AATGCGGCCGCTTTTCTTAAATC TTTGTTGC
U6	TCAGGTCATCACTATCGGCAAT	AAAGAAAGGGTGTAAAACGCA

Table S3. Statistical Comparisons Between Experimental Groups.

Figure	Comparison	*P* Value	Statistical methods	
Figure 1B	-1d Vehicle *v.s.* OXA	>0.9999	two-way repeated measures ANOVA followed by post hoc Bonferroni test	$F_{(4, 84)}$=17.96, P<0.0001
	3d Vehicle *v.s.* OXA	>0.9999		
	7d Vehicle *v.s.* OXA	0.0564		
	14d Vehicle *v.s.* OXA	<0.0001		
	21d Vehicle *v.s.* OXA	<0.0001		
Figure 1C	-1d Vehicle *v.s.* OXA	>0.9999	two-way repeated measures ANOVA followed by post hoc Bonferroni test	$F_{(4, 84)}$=4.497, P=0.0024
	3d Vehicle *v.s.* OXA	>0.9999		
	7d Vehicle *v.s.* OXA	0.1458		
	14d Vehicle *v.s.* OXA	0.0002		
	21d Vehicle *v.s.* OXA	0.0012		
Figure 1D	-1d Vehicle *v.s.* OXA	>0.9999		

	3d Vehicle *v.s.* OXA	0.9153	two-way repeated measures ANOVA followed by post hoc Bonferroni test	$F_{(4, 84)}$=16.10, P<0.0001
	7d Vehicle *v.s.* OXA	0.0006		
	14d Vehicle *v.s.* OXA	<0.0001		
	21d Vehicle *v.s.* OXA	<0.0001		
Figure 1E	-1d Vehicle *v.s.* OXA	0.5250	two-way repeated measures ANOVA followed by post hoc Bonferroni test	$F_{(4, 84)}$=9.763, P<0.0001
	3d Vehicle *v.s.* OXA	>0.9999		
	7d Vehicle *v.s.* OXA	0.0544		
	14d Vehicle *v.s.* OXA	0.1133		
	21d Vehicle *v.s.* OXA	<0.0001		
Figure 1F	-1d Vehicle *v.s.* OXA	0.7699	two-way repeated measures ANOVA followed by post hoc Bonferroni test	$F_{(4, 84)}$=5.699, P=0.0004
	3d Vehicle *v.s.* OXA	<0.0001		
	7d Vehicle *v.s.* OXA	<0.0001		
	14d Vehicle *v.s.* OXA	0.0002		
	21d Vehicle *v.s.* OXA	<0.0001		
Figure 1H	Vehicle *v.s.* OXA	0.0203	unpaired two-tailed Student's *t*-test.	t=2.674,df=12
Figure 1I	Vehicle *v.s.* OXA	0.0180	unpaired two-tailed Student's *t*-test.	t=2.707,df=13
Figure 2E	AAV-eGfp *v.s.* AAV-Cre	0.0016	unpaired two-tailed Student's *t*-test.	t=6.191,df=5
Figure 2F	AAV-eGfp *v.s.* AAV-Cre	0.0219	unpaired two-tailed Student's *t*-test.	t=2.712,df=10
Figure 2G	-1d AAV-eGfp *v.s.* AAV-Cre	>0.9999	two-way repeated measures ANOVA followed by post hoc Bonferroni test	$F_{(4, 144)}$=5.699, P<0.0001
	7d AAV-eGfp *v.s.* AAV-Cre	0.0001		
	14d AAV-eGfp *v.s.* AAV-Cre	<0.0001		
	21d AAV-eGfp *v.s.* AAV-Cre	0.0018		
	28d AAV-eGfp *v.s.* AAV-Cre	0.0001		
Figure 2H	-1d AAV-eGfp *v.s.* AAV-Cre	>0.9999	two-way repeated measures ANOVA followed by post hoc Bonferroni test	$F_{(4, 144)}$=0.3673, P<0.8316
	7d AAV-eGfp *v.s.* AAV-Cre	>0.9999		
	14d AAV-eGfp *v.s.* AAV-Cre	0.8070		
	21d AAV-eGfp *v.s.* AAV-Cre	>0.9999		
	28d AAV-eGfp *v.s.* AAV-Cre	0.5209		
Figure 2I	-1d AAV-eGfp *v.s.* AAV-Cre	0.1634	two-way repeated measures ANOVA followed by post hoc Bonferroni test	$F_{(4, 144)}$=22.09, P<0.0001
	7d AAV-eGfp *v.s.* AAV-Cre	<0.0001		
	14d AAV-eGfp *v.s.* AAV-Cre	<0.0001		
	21d AAV-eGfp *v.s.* AAV-Cre	<0.0001		
	28d AAV-eGfp *v.s.* AAV-Cre	<0.0001		
Figure 2J	-1d AAV-eGfp *v.s.* AAV-Cre	0.1086	two-way repeated measures ANOVA followed by post hoc Bonferroni test	$F_{(4, 144)}$=2.363, P<0.0558
	7d AAV-eGfp *v.s.* AAV-Cre	>0.9999		
	14d AAV-eGfp *v.s.* AAV-Cre	0.7195		
	21d AAV-eGfp *v.s.* AAV-Cre	>0.9999		
	28d AAV-eGfp *v.s.* AAV-Cre	0.0804		
Figure 2K	-1d AAV-eGfp *v.s.* AAV-Cre	0.8915		

	7d AAV-eGfp *v.s.* AAV-Cre	0.0054	two-way repeated measures ANOVA followed by post hoc Bonferroni test	$F_{(4, 144)}$=16.17, P<0.0001
	14d AAV-eGfp *v.s.* AAV-Cre	<0.0001		
	21d AAV-eGfp *v.s.* AAV-Cre	<0.0001		
	28d AAV-eGfp *v.s.* AAV-Cre	<0.0001		
Figure 3C	Naive *v.s.* Glucose+TET1-Control	0.9665	unpaired two-tailed Student's *t*-test.	t=0.04375,df=6
	Naive *v.s.* OXA+TET1-Control	0.0044		t=4.437,df=6
	Naive *v.s.* Glucose+TET1-Activation	0.1555		t=1.624,df=6
	Naive *v.s.* OXA+TET1-Activation	0.2362		t=1.316,df=6
	Glucose+TET1-Control *v.s.* OXA+TET1-Control	0.1486		t=1.657,df=6
	Glucose+TET1-Control *v.s.* Glucose+TET1-Activation	0.2111		t=1.400,df=6
	Glucose+TET1-Control *v.s.* OXA+TET1-Activation	0.3013		t=1.131,df=6
	OXA+TET1-Control *v.s.* Glucose+TET1-Activation	0.0307		t=2.812,df=6
	OXA+TET1-Control *v.s.* OXA+TET1-Activation	0.0443		t=2.536,df=6
	Glucose+TET1-Activation *v..s* OXA+TET1-Activation	0.8092		t=0.2523,df=6
Figure 3D	OXA+TET1-Control *v.s.* OXA+TET1-Activation	0.0346	unpaired two-tailed Student's *t*-test.	t=2.615,df=7
Figure 3F	-14d Naive *v.s.* Veh+ TET1-Control	0.1179	two-way repeated measures ANOVA followed by post hoc Bonferroni test	$F_{(20, 380)}$=5.213, P<0.0001
	-14d Naive *v.s.* OXA+ TET1-Control	0.0805		
	-14d Naive *v.s.* Veh+ TET1-OE	0.5712		
	-14d Naive *v.s.* OXA+ TET1-OE	>0.9999		
	-7d Naive *v.s.* Veh+ TET1-Control	>0.9999		
	-7d Naive *v.s.* OXA+ TET1-Control	>0.9999		
	-7d Naive *v.s.* Veh+ TET1-OE	0.9880		
	-7d Naive *v.s.* OXA+ TET1-OE	>0.9999		
	0d Naive *v.s.* Veh+ TET1-Control	0.8057		
	0d Naive *v.s.* OXA+ TET1-Control	>0.9999		
	0d Naive *v.s.* Veh+ TET1-OE	>0.9999		

	0d Naive *v.s.* OXA+ TET1-OE	0.5704		
	7d Naive *v.s.* Veh+ TET1-Control	>0.9999		
	7d Naive *v.s.* OXA+ TET1-Control	0.3226		
	7d Naive *v.s.* Veh+ TET1-OE	>0.9999		
	7d Naive *v.s.* OXA+ TET1-OE	>0.9999		
	14d Naive *v.s.* Veh+ TET1-Control	>0.9999		
	14d Naive *v.s.* OXA+ TET1-Control	***P<0.0001		
	14d Naive *v.s.* Veh+ TET1-OE	0.1829		
	14d Naive *v.s.* OXA+ TET1-OE	0.5286		
	21d Naive *v.s.* Veh+ TET1-Control	>0.9999		
	21d Naive *v.s.* OXA+ TET1-Control	***P<0.0001		
	21d Naive *v.s.* Veh+ TET1-OE	>0.9999		
	21d Naive *v.s.* OXA+ TET1-OE	>0.9999		
	-14d OXA+ TET1-Control *v.s.* OXA+ TET1-OE	0.3927	two-way repeated measures ANOVA followed by post hoc Bonferroni test	$F_{(5, 160)}$=8.630, P<0.0001
	-7d OXA+ TET1-Control *v.s.* OXA+ TET1-OE	>0.9999		
	0d OXA+ TET1-Control *v.s.* OXA+ TET1-OE	>0.9999		
	7d OXA+ TET1-Control *v.s.* OXA+ TET1-OE	>0.9999		
	14d OXA+ TET1-Control *v.s.* OXA+ TET1-OE	##P=0.0022		
	21d OXA+ TET1-Control *v.s.* OXA+ TET1-OE	###P=0.0006		
Figure 3G	-14d Naive *v.s.* Veh+ TET1-Control	>0.9999	two-way repeated measures ANOVA followed by post hoc Bonferroni test	$F_{(20, 380)}$=6.493 P<0.0001
	-14d Naive *v.s.* OXA+ TET1-Control	0.2827		
	-14d Naive *v.s.* Veh+ TET1-OE	0.1573		
	-14d Naive *v.s.* OXA+ TET1-OE	0.6017		
	-7d Naive *v.s.* Veh+ TET1-Control	>0.9999		

	-7d Naive *v.s.* OXA+ TET1-Control	0.1941		
	-7d Naive *v.s.* Veh+ TET1-OE	>0.9999		
	-7d Naive *v.s.* OXA+ TET1-OE	>0.9999		
	0d Naive *v.s.* Veh+ TET1-Control	>0.9999		
	0d Naive *v.s.* OXA+ TET1-Control	>0.9999		
	0d Naive *v.s.* Veh+ TET1-OE	>0.9999		
	0d Naive *v.s.* OXA+ TET1-OE	0.6650		
	7d Naive *v.s.* Veh+ TET1-Control	>0.9999		
	7d Naive *v.s.* OXA+ TET1-Control	$^{**}P$=0.0014		
	7d Naive *v.s.* Veh+ TET1-OE	>0.9999		
	7d Naive *v.s.* OXA+ TET1-OE	$^{*}P$=0.0488		
	14d Naive *v.s.* Veh+ TET1-Control	>0.9999		
	14d Naive *v.s.* OXA+ TET1-Control	$^{***}P$<0.0001		
	14d Naive *v.s.* Veh+ TET1-OE	>0.9999		
	14d Naive *v.s.* OXA+ TET1-OE	$^{***}P$<0.0001		
	21d Naive *v.s.* Veh+ TET1-Control	>0.9999		
	21d Naive *v.s.* OXA+ TET1-Control	$^{***}P$<0.0001		
	21d Naive *v.s.* Veh+ TET1-OE	>0.9999		
	21d Naive *v.s.* OXA+ TET1-OE	$^{***}P$<0.0001		
	-14d OXA+ TET1-Control *v.s.* OXA+ TET1-OE	>0.9999	two-way repeated measures ANOVA followed by post hoc Bonferroni test	$F_{(5, 160)}$=1.710 P=0.1353
	-7d OXA+ TET1-Control *v.s.* OXA+ TET1-OE	0.4303		
	0d OXA+ TET1-Control *v.s.* OXA+ TET1-OE	0.9349		
	7d OXA+ TET1-Control *v.s.* OXA+ TET1-OE	0.6026		
	14d OXA+ TET1-Control *v.s.* OXA+ TET1-OE	>0.9999		
	21d OXA+ TET1-Control *v.s.* OXA+ TET1-OE	>0.9999		

Figure 3H	-14d Naive *v.s.* Veh+ TET1-Control	>0.9999	two-way repeated measures ANOVA followed by post hoc Bonferroni test	$F_{(20, 380)}$=11.56, P<0.0001
	-14d Naive *v.s.* OXA+ TET1-Control	>0.9999		
	-14d Naive *v.s.* Veh+ TET1-OE	>0.9999		
	-14d Naive *v.s.* OXA+ TET1-OE	0.4935		
	-7d Naive *v.s.* Veh+ TET1-Control	0.7244		
	-7d Naive *v.s.* OXA+ TET1-Control	0.0626		
	-7d Naive *v.s.* Veh+ TET1-OE	0.2142		
	-7d Naive *v.s.* OXA+ TET1-OE	0.3008		
	0d Naive *v.s.* Veh+ TET1-Control	0.1197		
	0d Naive *v.s.* OXA+ TET1-Control	0.1871		
	0d Naive *v.s.* Veh+ TET1-OE	0.1988		
	0d Naive *v.s.* OXA+ TET1-OE	0.4120		
	7d Naive *v.s.* Veh+ TET1-Control	>0.9999		
	7d Naive *v.s.* OXA+ TET1-Control	0.1159		
	7d Naive *v.s.* Veh+ TET1-OE	>0.9999		
	7d Naive *v.s.* OXA+ TET1-OE	>0.9999		
	14d Naive *v.s.* Veh+ TET1-Control	0.3665		
	14d Naive *v.s.* OXA+ TET1-Control	$^{***}P$<0.0001		
	14d Naive *v.s.* Veh+ TET1-OE	0.0663		
	14d Naive *v.s.* OXA+ TET1-OE	$^{**}P$=0.0031		
	21d Naive *v.s.* Veh+ TET1-Control	>0.9999		
	21d Naive *v.s.* OXA+ TET1-Control	$^{***}P$<0.0001		
	21d Naive *v.s.* Veh+ TET1-OE	0.5885		
	21d Naive *v.s.* OXA+ TET1-OE	$^{*}P$=0.0463		
	-14d OXA+ TET1-Control *v.s.* OXA+ TET1-OE	>0.9999	two-way repeated measures ANOVA followed by post hoc Bonferroni test	$F_{(5, 160)}$=19.01 P<0.0001
	-7d OXA+ TET1-Control *v.s.* OXA+ TET1-OE	>0.9999		

	0d OXA+ TET1-Control *v.s.* OXA+ TET1-OE	>0.9999		
	7d OXA+ TET1-Control *v.s.* OXA+ TET1-OE	>0.9999		
	14d OXA+ TET1-Control *v.s.* OXA+ TET1-OE	###P<0.0001		
	21d OXA+ TET1-Control *v.s.* OXA+ TET1-OE	###P<0.0001		
Figure 3I	-14d Naive *v.s.* Veh+ TET1-Control	0.7644	two-way repeated measures ANOVA followed by post hoc Bonferroni test	$F_{(20, 380)}$=14.13, P<0.0001
	-14d Naive *v.s.* OXA+ TET1-Control	>0.9999		
	-14d Naive *v.s.* Veh+ TET1-OE	0.9862		
	-14d Naive *v.s.* OXA+ TET1-OE	>0.9999		
	-7d Naive *v.s.* Veh+ TET1-Control	>0.9999		
	-7d Naive *v.s.* OXA+ TET1-Control	0.3456		
	-7d Naive *v.s.* Veh+ TET1-OE	>0.9999		
	-7d Naive *v.s.* OXA+ TET1-OE	0.7854		
	0d Naive *v.s.* Veh+ TET1-Control	>0.9999		
	0d Naive *v.s.* OXA+ TET1-Control	0.5213		
	0d Naive *v.s.* Veh+ TET1-OE	>0.9999		
	0d Naive *v.s.* OXA+ TET1-OE	0.7153		
	7d Naive *v.s.* Veh+ TET1-Control	>0.9999		
	7d Naive *v.s.* OXA+ TET1-Control	0.1568		
	7d Naive *v.s.* Veh+ TET1-OE	>0.9999		
	7d Naive *v.s.* OXA+ TET1-OE	0.2819		
	14d Naive *v.s.* Veh+ TET1-Control	>0.9999		
	14d Naive *v.s.* OXA+ TET1-Control	***P<0.0001		
	14d Naive *v.s.* Veh+ TET1-OE	>0.9999		
	14d Naive *v.s.* OXA+ TET1-OE	***P<0.0001		
	21d Naive *v.s.* Veh+ TET1-Control	0.6179		

	21d Naive *v.s.* OXA+ TET1-Control	$^{***}P<0.0001$		
	21d Naive *v.s.* Veh+ TET1-OE	>0.9999		
	21d Naive *v.s.* OXA+ TET1-OE	$^{***}P<0.0001$		
	-14d OXA+ TET1-Control *v.s.* OXA+ TET1-OE	>0.9999	two-way repeated measures ANOVA followed by post hoc Bonferroni test	$F_{(5, 160)}$=1.214, P=0.3050
	-7d OXA+ TET1-Control *v.s.* OXA+ TET1-OE	>0.9999		
	0d OXA+ TET1-Control *v.s.* OXA+ TET1-OE	>0.9999		
	7d OXA+ TET1-Control *v.s.* OXA+ TET1-OE	>0.9999		
	14d OXA+ TET1-Control *v.s.* OXA+ TET1-OE	0.1440		
	21d OXA+ TET1-Control *v.s.* OXA+ TET1-OE	>0.9999		
Figure 3J	-14d Naive *v.s.* Veh+ TET1-Control	0.1442	two-way repeated measures ANOVA followed by post hoc Bonferroni test	$F_{(20, 380)}$=9.214, P<0.0001
	-14d Naive *v.s.* OXA+ TET1-Control	>0.9999		
	-14d Naive *v.s.* Veh+ TET1-OE	>0.9999		
	-14d Naive *v.s.* OXA+ TET1-OE	0.3355		
	-7d Naive *v.s.* Veh+ TET1-Control	0.0546		
	-7d Naive *v.s.* OXA+ TET1-Control	0.1365		
	-7d Naive *v.s.* Veh+ TET1-OE	0.0545		
	-7d Naive *v.s.* OXA+ TET1-OE	0.0689		
	0d Naive *v.s.* Veh+ TET1-Control	0.1171		
	0d Naive *v.s.* OXA+ TET1-Control	0.1233		
	0d Naive *v.s.* Veh+ TET1-OE	0.2911		
	0d Naive *v.s.* OXA+ TET1-OE	0.1327		
	7d Naive *v.s.* Veh+ TET1-Control	0.1561		
	7d Naive *v.s.* OXA+ TET1-Control	$^{**}P$=0.0059		
	7d Naive *v.s.* Veh+ TET1-OE	0.5135		
	7d Naive *v.s.* OXA+ TET1-OE	$^{*}P$=0.0259		

	14d Naive *v.s.* Veh+ TET1-Control	0.1339		
	14d Naive *v.s.* OXA+ TET1-Control	$^{***}P<0.0001$		
	14d Naive *v.s.* Veh+ TET1-OE	0.9822		
	14d Naive *v.s.* OXA+ TET1-OE	$^{***}P<0.0001$		
	21d Naive *v.s.* Veh+ TET1-Control	>0.9999		
	21d Naive *v.s.* OXA+ TET1-Control	$^{***}P<0.0001$		
	21d Naive *v.s.* Veh+ TET1-OE	>0.9999		
	21d Naive *v.s.* OXA+ TET1-OE	0.8593		
	-14d OXA+ TET1-Control *v.s.* OXA+ TET1-OE	>0.9999	two-way repeated measures ANOVA followed by post hoc Bonferroni test	$F_{(5, 160)}=13.71$, $P<0.0001$
	-7d OXA+ TET1-Control *v.s.* OXA+ TET1-OE	>0.9999		
	0d OXA+ TET1-Control *v.s.* OXA+ TET1-OE	>0.9999		
	7d OXA+ TET1-Control *v.s.* OXA+ TET1-OE	>0.9999		
	14d OXA+ TET1-Control *v.s.* OXA+ TET1-OE	$^{\#\#\#}P<0.0001$		
	21d OXA+ TET1-Control *v.s.* OXA+ TET1-OE	$^{\#\#\#}P<0.0001$		
Figure 4B	Vehicle *v.s.* OXA	0.0127	unpaired two-tailed Student's *t*-test	$t=2.631, df=34$
Figure 4C	Vehicle *v.s.* OXA	0.1723	unpaired two-tailed Student's *t*-test	$t=1.393, df=35$
Figure 4D	Vehicle *v.s.* OXA	0.0009	unpaired two-tailed Student's *t*-test	$t=3.632, df=34$
Figure 4E	Vehicle *v.s.* OXA	0.1700	unpaired two-tailed Student's *t*-test	$t=1.402, df=34$
Figure 4F	Vehicle *v.s.* OXA (1× Rheobase)	0.6466	unpaired two-tailed Mann-Whitney U test	Mann-Whitney U =140.5
	Vehicle *v.s.* OXA (2× Rheobase)	0.0117	unpaired two-tailed Mann-Whitney U test	Mann-Whitney U =89.50
Figure 4H	AAV-eGfp *v.s.* AAV-Cre	0.3757	unpaired two-tailed Student's *t*-test	$t=0.8935, df=52$
Figure 4I	AAV-eGfp *v.s.* AAV-Cre	0.0756	unpaired two-tailed Student's *t*-test	$t=1.811, df=56$

Figure 4J	AAV-eGfp *v.s.* AAV-Cre	0.0027	unpaired two-tailed Student's *t*-test	t=3.139,df=58
Figure 4K	AAV-eGfp *v.s.* AAV-Cre	0.6982	unpaired two-tailed Student's *t*-test	t=0.3896,df=58
Figure 4L	AAV-eGfp *v.s.* AAV-Cre (1× Rheobase)	0.8681	unpaired two-tailed Mann-Whitney U test	Mann-Whitney U =392
	AAV-eGfp *v.s.* AAV-Cre (2× Rheobase)	0.0248	unpaired two-tailed Mann-Whitney U test	Mann-Whitney U =249.5
Figure 4N	OXA+TET1-Control *v.s.* OXA+TET1-OE	0.0058	unpaired two-tailed Student's *t*-test	t=2.939,df=35
Figure 4O	OXA+TET1-Control *v.s.* OXA+TET1-OE	0.0821	unpaired two-tailed Student's *t*-test	t=1.792,df=34
Figure 4P	OXA+TET1-Control *v.s.* OXA+TET1-OE	0.0302	unpaired two-tailed Student's *t*-test	t=2.253,df=37
Figure 4Q	OXA+TET1-Control *v.s.* OXA+TET1-OE	0.4403	unpaired two-tailed Student's *t*-test	t=0.7801,df=37
Figure 4R	OXA+TET1-Control *v.s.* OXA+TET1-OE (1× Rheobase)	0.8827	unpaired two-tailed Mann-Whitney U test	Mann-Whitney U =156
	OXA+TET1-Control *v.s.* OXA+TET1-OE (2× Rheobase)	0.9795	unpaired two-tailed Mann-Whitney U test	Mann-Whitney U =119
Figure 5A	*Scn3a*-Vehicle *v.s.* *Scn3a*-OXA	0.3437	unpaired two-tailed Student's *t*-test	t=0.9894,df=11
	Scn8a-Vehicle *v.s.* *Scn8a*-OXA	0.0207		t=2.606,df=14
	Scn9a-Vehicle *v.s.* *Scn9a*-OXA	0.3397		t=1.003,df=10
	Scn10a-Vehicle *v.s.* *Scn10a*-OXA	0.4533		t=0.7731,df=13
	Scn11a-Vehicle *v.s.* *Scn11a*-OXA	0.3783		t=0.9179,df=11
Figure 5C	Vehicle *v.s.* OXA	0.0225	unpaired two-tailed Student's *t*-test	t=2.819,df=8
Figure 6B	AAV-eGfp *v.s.* AAV-Cre	0.0377	unpaired two-tailed Student's *t*-test	t=3.060,df=4
Figure 6C	AAV-eGfp *v.s.* AAV-Cre	0.0286	unpaired two-tailed Kolmogorov-Smirnov test	Kolmogorov-Smirnov D=1.000
Figure 6E	Vehicle *v.s.* OXA+TET1-Control	0.0143	one-way repeated measures ANOVA followed by post hoc Tukey test	$F_{(2, 15)}$=6.353, P=0.0100
	Vehicle *v.s.* OXA+TET1-OE	0.9414		
	OXA+TET1-Control *v.s.* OXA+TET1-OE	0.0275		
Figure 6F	Vehicle *v.s.* OXA+TET1-Control	0.0246	one-way repeated measures ANOVA	$F_{(2, 11)}$=6.864, P=0.0116

	Vehicle *v.s.* OXA+TET1-OE	>0.9999	followed by post hoc Bonferroni test	
	OXA+TET1-Control *v.s.* OXA+TET1-OE	0.0284		
Figure 6G	Vehicle *v.s.* OXA+TET1-Control	0.5416	one-way repeated measures ANOVA followed by post hoc Tukey test	$F_{(2, 37)}$=3.201, *P*=0.0522
	Vehicle *v.s.* OXA+TET1-OE	0.2682		
	OXA+TET1-Control *v.s.* OXA+TET1-OE	0.0464		
Figure 6I	Vehicle *v.s.* OXA+TET1-Control	0.3750	one-way repeated measures ANOVA followed by post hoc Tukey test	$F_{(2, 19)}$=1.012, *P*=0.3824
	Vehicle *v.s.* OXA+TET1-OE	0.6370		
	OXA+TET1-Control *v.s.* OXA+TET1-OE	0.8863		
Figure 6K	Vehicle *v.s.* OXA+TET1-Control	0.5169	one-way repeated measures ANOVA followed by post hoc Tukey test	$F_{(2, 14)}$=4.290, *P*=0.0352
	Vehicle *v.s.* OXA+TET1-OE	0.2562		
	OXA+TET1-Control *v.s.* OXA+TET1-OE	0.0287		
Figure 6M	Vehicle *v.s.* OXA+TET1-Control	0.7531	one-way repeated measures ANOVA followed by post hoc Tukey test	$F_{(2, 15)}$=0.8087, *P*=0.4639
	Vehicle *v.s.* OXA+TET1-OE	0.4341		
	OXA+TET1-Control *v.s.* OXA+TET1-OE	0.8515		
Figure 7A	Vehicle *v.s.* OXA	0.0088	unpaired two-tailed Student's *t*-test	*t*=3.012,*df*=15
Figure 7B	AAV-eGfp *v.s.* AAV-Cre	0.0969	unpaired two-tailed Student's *t*-test	*t*=2.160,*df*=4
Figure 7C	OXA+TET1-Control *v.s.* OXA+TET1-OE	0.0315	unpaired two-tailed Student's *t*-test	*t*=2.680,*df*=7
Figure 7D	Vehicle *v.s.* OXA	0.0309	unpaired two-tailed Student's *t*-test	*t*=2.806,*df*=6
Figure 7E	AAV-eGfp *v.s.* AAV-Cre	0.0407	unpaired two-tailed Student's *t*-test	*t*=2.438,*df*=8
Figure 7F	AAV-eGfp *v.s.* AAV-Cre	0.9773	unpaired two-tailed Student's *t*-test	*t*=0.02935,*df*=8
Figure 7G	Naive *v.s.* OXA+TET1-Control	0.0002	one-way repeated measures ANOVA followed by post hoc Dunnett test	$F_{(2, 11.36)}$=3.620, *P*=0.0001
	Naive *v.s.* OXA+TET1-OE	0.0148		
	OXA+TET1-Control *v.s.* OXA+TET1-OE	0.0007		
Figure 7I	Vehicle *v.s.* OXA	0.0047	unpaired two-tailed Student's *t*-test	*t*=3.619,*df*=10
Figure 7J	OXA+TET1-Control *v.s.* OXA+TET1-OE	0.0292	unpaired two-tailed Student's *t*-test	*t*=2.652,*df*=8

Figure 7M	OXA+miR-NC *v.s.* OXA+miR-30b agomir	0.0003	unpaired two-tailed Student's *t*-test	t=5.338,df=10
Figure 7N	-1d OXA+miR-NC *v.s.* OXA+miR-30b agomir	>0.9999	two-way repeated measures ANOVA followed by post hoc Bonferroni test	$F_{(5, 70)}$=2.285, P=0.0554
	1d OXA+miR-NC *v.s.* OXA+miR-30b agomir	>0.9999		
	3d OXA+miR-NC *v.s.* OXA+miR-30b agomir	>0.9999		
	5d OXA+miR-NC *v.s.* OXA+miR-30b agomir	>0.9999		
	7d OXA+miR-NC *v.s.* OXA+miR-30b agomir	>0.9999		
	14d OXA+miR-NC *v.s.* OXA+miR-30b agomir	0.0085		
Figure 7P	OXA *v.s.* OXA+TET1-OE+miR-NC	0.0006	one-way repeated measures ANOVA followed by post hoc Tukey test	$F_{(2, 8)}$=20.22, P=0.0007
	OXA *v.s.* OXA+TET1-OE+miR-30b antagomir	0.0514		
	OXA+TET1-OE+miR-NC *v.s.* OXA+TET1-OE+miR-30b antagomir	0.0378		
Figure 7Q	-1d OXA *v.s.* OXA+TET1-OE+miR-NC	>0.9999	two-way repeated measures ANOVA followed by post hoc Bonferroni test	$F_{(10, 105)}$=2.355, P=0.0149
	-1d OXA *v.s.* OXA+TET1-OE+miR-30b antagomir	>0.9999		
	-1d OXA+TET1-OE+miR-NC *v.s.* OXA+TET1-OE+miR-30b antagomir	>0.9999		
	1d OXA *v.s.* OXA+TET1-OE+miR-NC	0.9992		
	1d OXA *v.s.* OXA+TET1-OE+miR-30b antagomir	>0.9999		
	1d OXA+TET1-OE+miR-NC *v.s.* OXA+TET1-OE+miR-30b antagomir	>0.9999		
	3d OXA *v.s.* OXA+TET1-OE+miR-NC	0.1141		
	3d OXA *v.s.* OXA+TET1-OE+miR-30b antagomir	0.1141		
	3d OXA+TET1-OE+miR-NC *v.s.* OXA+TET1-OE+miR-30b antagomir	>0.9999		
	5d OXA *v.s.* OXA+TET1-OE+miR-NC	0.7718		

	5d OXA *v.s.* OXA+TET1-OE+miR-30b antagomir	0.7718		
	5d OXA+TET1-OE+miR-NC *v.s.* OXA+TET1-OE+miR-30b antagomir	>0.9999		
	7d OXA *v.s.* OXA+TET1-OE+miR-NC	0.0002		
	7d OXA *v.s.* OXA+TET1-OE+miR-30b antagomir	0.0012		
	7d OXA+TET1-OE+miR-NC *v.s.* OXA+TET1-OE+miR-30b antagomir	>0.9999		
	14d OXA *v.s.* OXA+TET1-OE+miR-NC	0.0012		
	14d OXA *v.s.* OXA+TET1-OE+miR-30b antagomir	>0.9999		
	14d OXA+TET1-OE+miR-NC *v.s.* OXA+TET1-OE+miR-30b antagomir	0.0192		
Supporting information Figure S1	Vehicle *v.s.* OXA	0.3329	unpaired two-tailed Student's *t*-test	*t*=1.017,*df*=10
Supporting information Figure S2A	Vehicle *v.s.* OXA	0.2331	unpaired two-tailed Student's *t*-test	*t*=1.213,*df*=35
Supporting information Figure S2B	AAV-eGfp *v.s.* AAV-Cre	0.1227	unpaired two-tailed Student's *t*-test	*t*=1.567,*df*=56
Supporting information Figure S2C	OXA+TET1-Control *v.s.* OXA+TET1-OE	0.3792	unpaired two-tailed Student's *t*-test	*t*=0.8907,*df*=35
Supporting information Figure S3A	Vehicle *v.s.* OXA	0.2331	unpaired two-tailed Student's *t*-test	*t*=1.213,*df*=35
Supporting information Figure S3B	Vehicle *v.s.* OXA	0.1858	unpaired two-tailed Student's *t*-test	*t*=1.370,*df*=20
Supporting information Figure S3C	Vehicle *v.s.* OXA	0.2038	unpaired two-tailed Student's *t*-test	*t*=1.314,*df*=20
Supporting information Figure S3D	Vehicle *v.s.* OXA	0.1519	unpaired two-tailed Student's *t*-test	*t*=1.500,*df*=17

Supporting information Figure S3E	Vehicle *v.s.* OXA	0.3108	unpaired two-tailed Student's *t*-test	*t*=1.055,*df*=13
Supporting information Figure S3F	Vehicle *v.s.* OXA (1× Rheobase)	0.8081	unpaired two-tailed Mann-Whitney U test	Mann-Whitney U =54
	Vehicle *v.s.* OXA (2× Rheobase)	0.0141	unpaired two-tailed Mann-Whitney U test	Mann-Whitney U =23.5
Supporting information Figure S3G	AAV-eGfp *v.s.* AAV-Cre	0.1278	unpaired two-tailed Student's *t*-test	*t*=1.568,*df*=29
Supporting information Figure S3H	AAV-eGfp *v.s.* AAV-Cre	0.5837	unpaired two-tailed Student's *t*-test	*t*=0.5547,*df*=27
Supporting information Figure S3I	AAV-eGfp *v.s.* AAV-Cre	0.0005	unpaired two-tailed Student's *t*-test	*t*=3.928,*df*=29
Supporting information Figure S3J	AAV-eGfp *v.s.* AAV-Cre	0.4839	unpaired two-tailed Student's *t*-test	*t*=0.7088,*df*=30
Supporting information Figure S3K	AAV-eGfp *v.s.* AAV-Cre	0.9835	unpaired two-tailed Student's *t*-test	*t*=0.02085,*df*=30
Supporting information Figure S3L	AAV-eGfp *v.s.* AAV-Cre (1× Rheobase)	>0.9999	unpaired two-tailed Mann-Whitney U test	Mann-Whitney U =119
	AAV-eGfp *v.s.* AAV-Cre (2× Rheobase)	0.2379	unpaired two-tailed Mann-Whitney U test	Mann-Whitney U =90
Supporting information Figure S3M	OXA+TET1-Control *v.s.* OXA+TET1-OE	0.7580	unpaired two-tailed Student's *t*-test	*t*=0.3124,*df*=20
Supporting information Figure S3N	OXA+TET1-Control *v.s.* OXA+TET1-OE	0.2332	unpaired two-tailed Student's *t*-test	*t*=1.239,*df*=16
Supporting information Figure S3O	OXA+TET1-Control *v.s.* OXA+TET1-OE	0.1718	unpaired two-tailed Student's *t*-test	*t*=1.417,*df*=20
Supporting information Figure S3P	OXA+TET1-Control *v.s.* OXA+TET1-OE	0.0274	unpaired two-tailed Student's *t*-test	*t*=2.400,*df*=18
Supporting information Figure S3Q	OXA+TET1-Control *v.s.* OXA+TET1-OE	0.5059	unpaired two-tailed Student's *t*-test	*t*=0.6788,*df*=18
	OXA+TET1-Control *v.s.* OXA+TET1-OE	>0.9999	unpaired two-tailed Mann-Whitney U test	Mann-Whitney U =55

Supporting information Figure S3R	OXA+TET1-Control *v.s.* OXA+TET1-OE	0.7752	unpaired two-tailed Mann-Whitney U test	Mann-Whitney U =55.5
Supporting information Figure S4A	Vehicle *v.s.* OXA	0.7747	unpaired two-tailed Student's *t*-test	t=0.2923,df=13
Supporting information Figure S4B	Vehicle *v.s.* OXA	0.5359	unpaired two-tailed Student's *t*-test	t=0.6359,df=13
Supporting information Figure S4C	Vehicle *v.s.* OXA	0.1233	unpaired two-tailed Student's *t*-test	t=1.648,df=13
Supporting information Figure S4D	Vehicle *v.s.* OXA	0.0013	unpaired two-tailed Student's *t*-test	t=3.934,df=15
Supporting information Figure S4E	Vehicle *v.s.* OXA	0.0337	unpaired two-tailed Student's *t*-test	t=2.290,df=19
Supporting information Figure S4F	Vehicle *v.s.* OXA (1× Rheobase)	0.1923	unpaired two-tailed Mann-Whitney U test	Mann-Whitney U =14
	Vehicle *v.s.* OXA (2× Rheobase)	0.6674	unpaired two-tailed Mann-Whitney U test	Mann-Whitney U =23
Supporting information Figure S4G	AAV-eGfp *v.s.* AAV-Cre	0.0891	unpaired two-tailed Student's *t*-test	t=1.769,df=25
Supporting information Figure S4H	AAV-eGfp *v.s.* AAV-Cre	0.6789	unpaired two-tailed Student's *t*-test	t=0.4193,df=23
Supporting information Figure S4I	AAV-eGfp *v.s.* AAV-Cre	0.2006	unpaired two-tailed Student's *t*-test	t=1.314,df=25
Supporting information Figure S4J	AAV-eGfp *v.s.* AAV-Cre	0.0008	unpaired two-tailed Student's *t*-test	t=3.803,df=26
Supporting information Figure S4K	AAV-eGfp *v.s.* AAV-Cre	0.5176	unpaired two-tailed Student's *t*-test	t=0.6561,df=26
Supporting information Figure S4L	AAV-eGfp *v.s.* AAV-Cre (1× Rheobase)	>0.9999	unpaired two-tailed Mann-Whitney U test	Mann-Whitney U =78
	AAV-eGfp *v.s.* AAV-Cre (2× Rheobase)	0.0473	unpaired two-tailed Mann-Whitney U test	Mann-Whitney U =40
Supporting information	OXA+TET1-Control *v.s.* OXA+TET1-OE	0.0525	unpaired two-tailed Student's *t*-test	t=2.134,df=13

Figure S4M				
Supporting information Figure S4N	OXA+TET1-Control *v.s.* OXA+TET1-OE	0.0182	unpaired two-tailed Student's *t*-test	t=2.614,df=17
Supporting information Figure S4O	OXA+TET1-Control *v.s.* OXA+TET1-OE	0.0261	unpaired two-tailed Student's *t*-test	t=2.537,df=12
Supporting information Figure S4P	OXA+TET1-Control *v.s.* OXA+TET1-OE	0.1519	unpaired two-tailed Student's *t*-test	t=1.500,df=17
Supporting information Figure S4Q	OXA+TET1-Control *v.s.* OXA+TET1-OE	0.5734	unpaired two-tailed Student's *t*-test	t=0.5741,df=17
Supporting information Figure S4R	OXA+TET1-Control *v.s.* OXA+TET1-OE (1× Rheobase)	>0.9999	unpaired two-tailed Mann-Whitney U test	Mann-Whitney U =26
	OXA+TET1-Control *v.s.* OXA+TET1-OE (2× Rheobase)	>0.9999	unpaired two-tailed Mann-Whitney U test	Mann-Whitney U =12.5
Supporting information Figure S5A	Naive *v.s.* OXA+TET1-Control	0.5140	one-way repeated measures ANOVA followed by post hoc Tukey test	$F_{(2, 37)}$=0.7888, P=0.4619
	Naive *v.s.* OXA+TET1-OE	0.9995		
	OXA+TET1-Control *v.s.* OXA+TET1-OE	0.4983		
Supporting information Figure S5C	Vehicle *v.s.* OXA+TET1-Control	0.8138	one-way repeated measures ANOVA followed by post hoc Tukey test	$F_{(2, 20)}$=0.7650, P=0.4785
	Vehicle *v.s.* OXA+TET1-OE	0.4478		
	OXA+TET1-Control *v.s.* OXA+TET1-OE	0.8673		

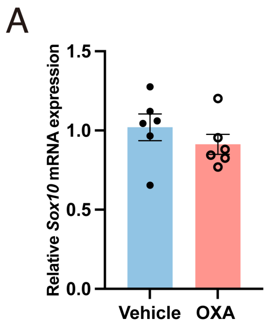

Figure S1

Oxaliplatin treatment does not affect the mRNA levels of *Sox10* in the mouse DRG.

(A): The mRNA levels of *Sox10* in the DRG of the Vehicle group and the OXA group (unpaired *t*-test. Each sample contained six DRG from one mouse, $n = 6$).

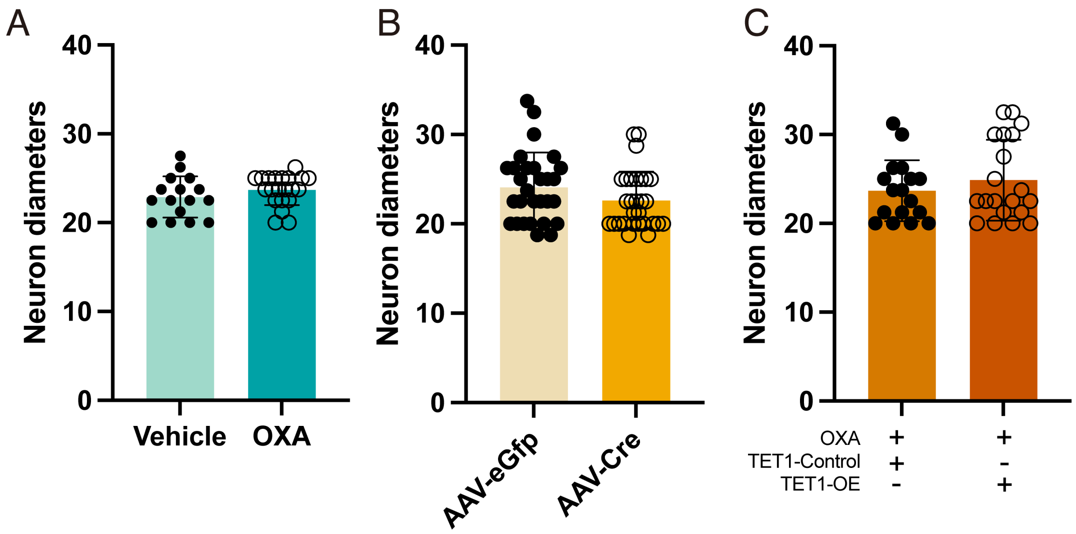

Figure S2

The diameters of neurons recording action potentials were similar across all groups.

(A): No differences were observed in the diameters of DRG neurons with recorded action potentials between the Vehicle and OXA groups (unpaired *t*-test. $n = 16$ in Vehicle, $n = 21$ in OXA). (B): No differences were observed in the diameters of DRG neurons with recorded action potentials between the AAV-eGfp and AAV-Cre groups (unpaired *t*-test. $n = 29$). (C): No differences were observed in the diameters of DRG neurons with recorded action potentials between the OXA+TET1-Control and OXA+TET1-OE groups (unpaired *t*-test. $n = 17$ in OXA+TET1-Control, $n = 20$ in OXA+TET1-OE).

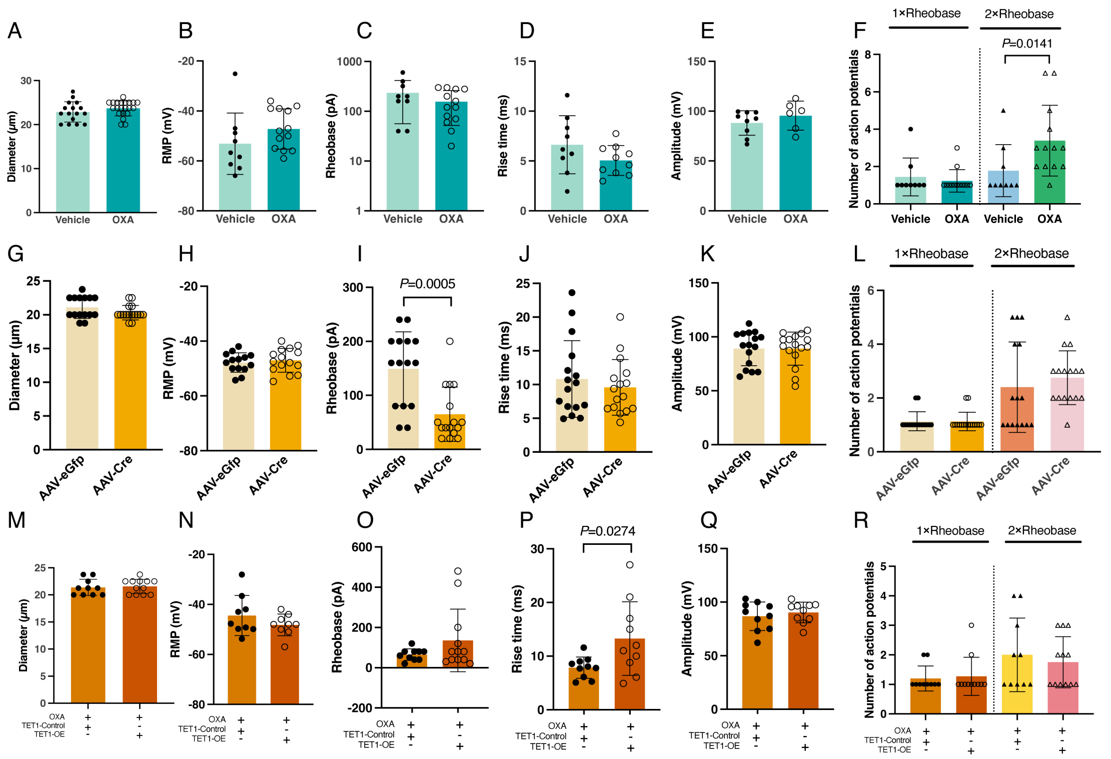

Figure S3

Action potentials in small-diameter neurons of the DRG after OXA treatment and modulation of TET1 expression.

(A): The diameter of each recorded neuron in the Vehicle and OXA groups (unpaired *t*-test. $n = 16$ in Vehicle, $n = 21$ in OXA). (B–E): There were no differences in RMP (B), Rheobase (C), Rise time (D), or Amplitude (E) between the Vehicle and OXA groups (unpaired *t*-test. n = 6-13). (F): The spike number at twice the rheobase in DRG neurons of the OXA group was increased compared to the Vehicle group (unpaired Mann Whitney test. n = 9-13). (G): The diameter of each recorded neuron in the AAV-eGfp and AAV-Cre groups (unpaired *t*-test. n = 15-16). (H): There were no differences in RMP between the AAV-eGfp and AAV-Cre groups (unpaired *t*-test. n = 14-15). (I): The rheobase was lower in the AAV-Cre group compared to the AAV-eGfp group (unpaired *t*-test. n = 15-16). (J–K): There were no differences in Rise time (J) or Amplitude (K) between the AAV-eGfp and AAV-Cre

groups (unpaired *t*-test. *n* = 16). (L): Spike counts at 1x and 2x rheobase in DRG neurons were consistent between the AAV-eGFP and AAV-Cre groups (unpaired Mann Whitney test. *n* = 15-16). (M): The diameter of each recorded neuron in the OXA+TET1-Control and OXA+TET1-OE groups (unpaired *t*-test. *n* = 10-12). (N–O): There were no differences in RMP (N) or Rheobase (O) between the OXA+TET1-Control and OXA+TET1-OE groups (unpaired *t*-test. *n* = 9-12). (P): The rise time was longer in the OXA+TET1-OE group compared to the OXA+TET1-Control group (unpaired *t*-test. *n* = 10). (Q): There were no differences in amplitude between the OXA+TET1-Control and OXA+TET1-OE groups (unpaired *t*-test. *n* = 10). (R): Spike counts at 1x and 2x rheobase in DRG neurons were consistent between the OXA+TET1-Control and OXA+TET1-OE groups (unpaired Mann Whitney test. *n* = 10-12).

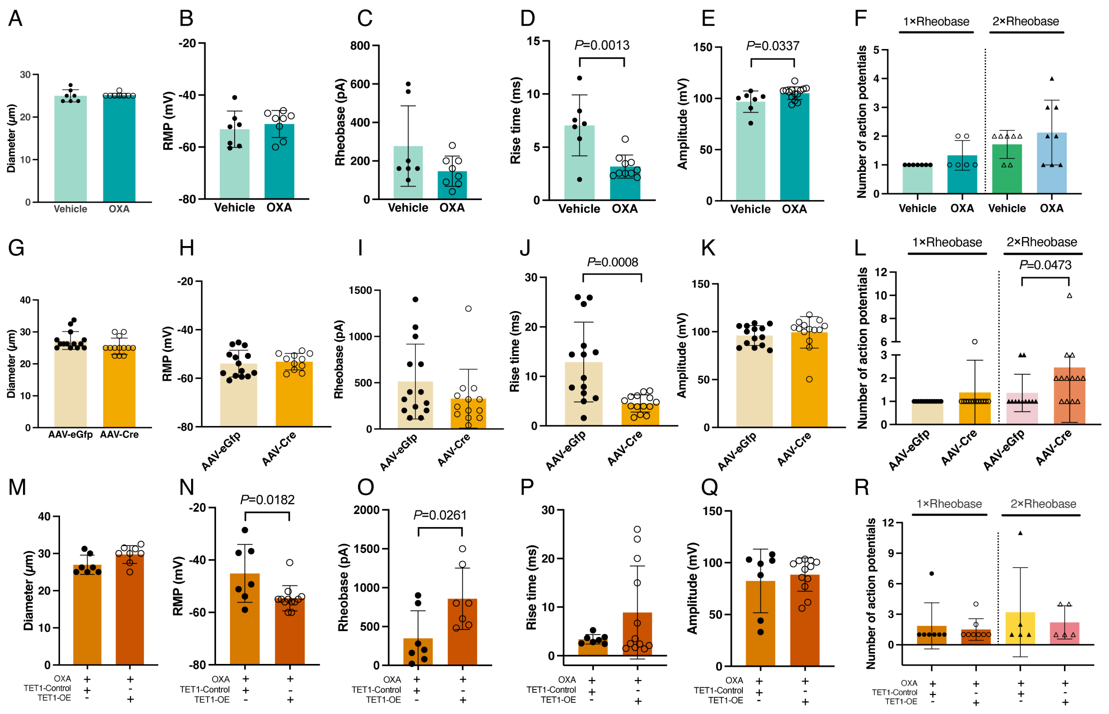

Figure S4

Action potentials in medium-diameter neurons of the DRG after OXA treatment and modulation of TET1 expression.

(A): The diameter of each recorded neuron in the Vehicle and OXA groups (unpaired *t*-test. n = 7-8). (B–C): There were no differences in RMP (B) or Rheobase (C) between the Vehicle and OXA groups (unpaired *t*-test. n = 7-8). (D–E): In the OXA group, the rise time (D) was reduced and the amplitude (E) increased compared to the Vehicle group (unpaired *t*-test. n = 7-14). (F): Spike counts at 1x and 2x rheobase in DRG neurons were consistent between the Vehicle and OXA groups (unpaired Mann Whitney test. n = 6-8). (G): The diameter of each recorded neuron in the AAV-eGfp and AAV-Cre groups (unpaired *t*-test. n = 13-14). (H–I): There were no differences in RMP (H) or Rheobase (I) between the AAV-eGfp and AAV-Cre groups (unpaired *t*-test. n = 11-14). (J): In the AAV-Cre group, the rise time was reduced and the amplitude increased compared to the AAV-eGfp group (unpaired *t*-test. n = 14). (K): There were no differences in amplitude between the AAV-eGfp and AAV-Cre groups (unpaired *t*-test. n = 14). (L): The spike number at twice the rheobase in DRG neurons of the AAV-Cre group was increased compared to the AAV-eGfp group (unpaired Mann Whitney test. n = 11-13). (M): The diameter of each recorded neuron in the OXA+TET1-Control and OXA+TET1-OE groups (unpaired *t*-test. n = 7-8). (N–O): In the OXA+TET1-OE group, the RMP (N) was reduced and the Rheobase (O) increased compared to the OXA+TET1-Control group (unpaired *t*-test. n = 7-12). (P–Q): There were no differences in rise time (P) or amplitude (Q) between the OXA+TET1-Control and OXA+TET1-OE groups (unpaired *t*-test. n = 7-12). (R): Spike counts at 1x and 2x rheobase in DRG neurons were consistent between the OXA+TET1-Control and OXA+TET1-OE groups (unpaired

Mann Whitney test. *n* = 5-8).

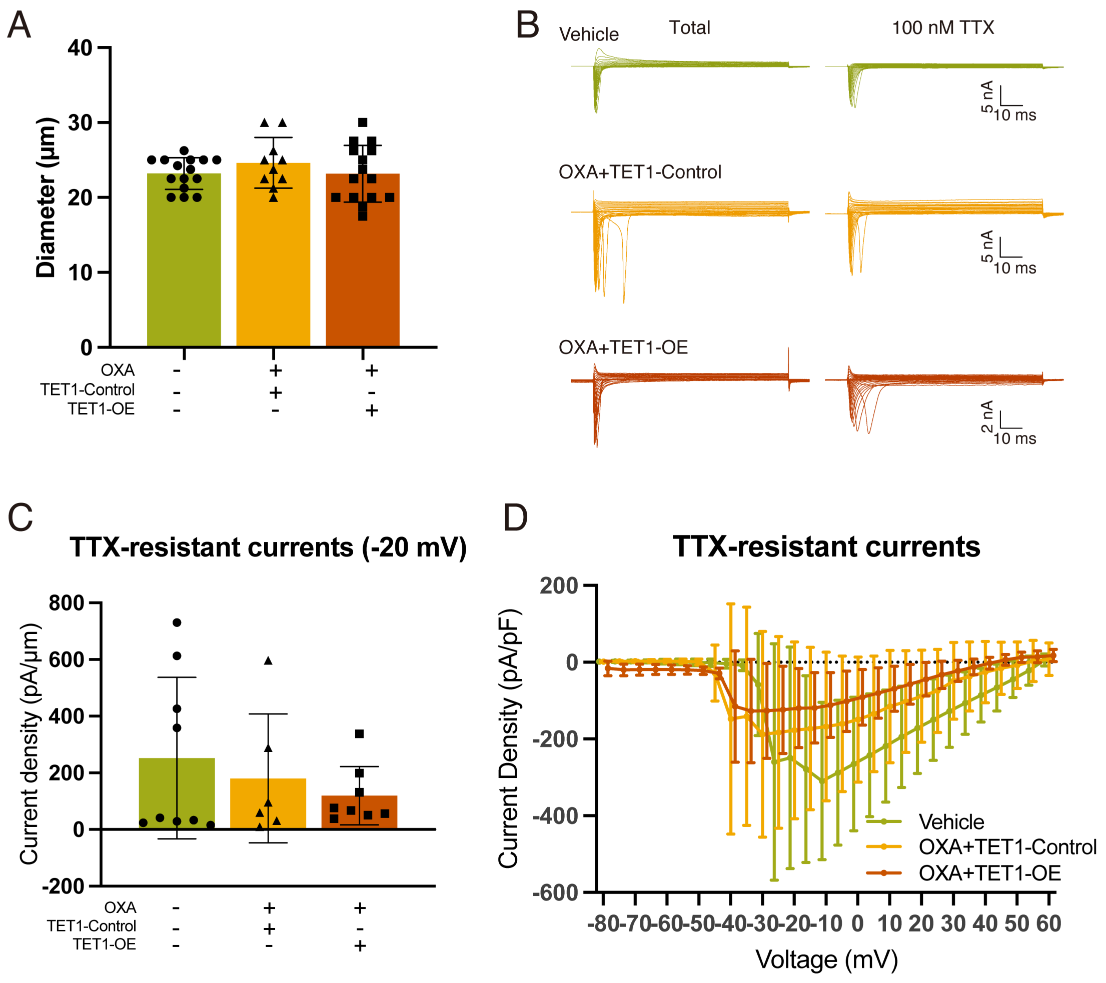

Figure S5

Neither oxaliplatin nor TET1 affects TTX-resistant currents in the DRG.

(A): The diameters of neurons recording action potentials in each group (one-way ANOVA. *n* = 15 in Veh and OXA+TET1-OE groups, and *n* = 10 in OXA+TET1-Control group). (B): Representative traces of total Nav and TTX-sensitive currents in DRG neurons. (C): There was no difference in TTX-resistant current density (-20mV) among the three groups (one-way ANOVA. *n* = 9 in Veh, *n* = 6 in OXA+TET1-Control group, *n* = 8 in OXA+TET1-OE group). (D): I-V curves of TTX-resistant currents exhibited a

leftward shift in both the OXA+TET1-Control and OXA+TET1-OE groups compared to the Vehicle group.
